# Processing of Distillery Stillage to Recover Phenolic Compounds with Ultrasound-Assisted and Microwave-Assisted Extractions

**DOI:** 10.3390/ijerph19052709

**Published:** 2022-02-25

**Authors:** Wioleta Mikucka, Magdalena Zielinska, Katarzyna Bulkowska, Izabela Witonska

**Affiliations:** 1 Department of Environmental Biotechnology, Faculty of Geoengineering, University of Warmia and Mazury in Olsztyn, Sloneczna St. 45G, 10-709 Olsztyn, Poland; magdalena.zielinska@uwm.edu.pl (M.Z.); katarzyna.bulkowska@uwm.edu.pl (K.B.); 2 Faculty of Chemistry, Institute of General and Ecological Chemistry, Lodz University of Technology, Zeromskiego St. 116, 90-924 Lodz, Poland; izabela.witonska@p.lodz.pl

**Keywords:** distillery by-product, bioactive compounds, phenolic acids, flavonoids, HPLC, waste valorization

## Abstract

This study investigated the effect of ultrasound-assisted extraction (UAE) and microwave-assisted extraction (MAE) conditions (extraction time, acetone concentration, solid-to-solvent ratio) on the efficiency of polyphenol recovery from distillery stillage and antioxidant activity of the extracts. The highest total polyphenol content, flavonoid content, and phenolic acid content were obtained with 10-min UAE and 5-min MAE at a solid-to-acetone ratio of 1:15 (*w*:*v*). Recovery yield was the highest with an aqueous solution of 60% acetone, confirming the results of Hansen Solubility Parameter analysis. Although UAE resulted in approximately 1.2 times higher extraction yield, MAE showed a better balance between extraction yield and energy consumption exhibited by its 3-fold higher extraction rate than that of UAE. Content of total polyphenols and phenolic acids strongly correlated with antioxidant activity, indicating that these compounds provide a substantial contribution to the bioactive properties of the extracts. Six phenolic acids were extracted, predominately ferulic and p-coumaric acids, and free forms of these acids constituted 91% of their total content, which opens various possibilities for their application in the food, cosmetics, and pharmaceutical industries.

## 1. Introduction

Sustainable industrial development and waste valorization have become key industry concerns. Distilleries are one of the most polluting industries since 88% of their raw materials are converted into distillery stillage, which is difficult to handle due to its low pH, high organic matter content, and dark brown color [1]. The utilization of the by-products from alcohol production is based on using them as animal feed [2], field fertilizer [1], and for biogas production [3]. However, such uses of stillage still fail to completely solve the problem of its management. Therefore, there is an emphasis on the circular economy concept in which the by-products remain in circulation for as long as possible. The employment of such an approach resulted in the development of processes that first ensure the recovery of valuable chemical compounds for the economy, and then valorization of the waste. Among the methods of stillage utilization, recovery of bioactive compounds represents a novel approach, which would not only reduce the pollution generated during alcohol production but also contribute to the economic competitiveness and sustainable development of the distillery industry.

Bioactive compounds present in plant-origin materials, such as waste from the distillery industry, include polyphenols [4]. Polyphenols are classified according to their structure as phenolic acids, flavonoids, and tannins. These molecules have an aromatic ring containing one or more hydroxyl groups; they function as hydrogen donors, antioxidants, and oxygen quenchers [5]. These functions render polyphenolic substances of great interest, owing to their role in preventing various human diseases [4,6]. Due to the biological properties, polyphenols have the potential to be used in food, pharmaceutical, cosmetics, and organic fine chemistry industries. These compounds find applications related to food dying, food preservation, bioactive packaging, improvement of the physicochemical properties of starch, and production of prebiotic ingredients, surfactants, cosmetic products, hydrogels and nanocomplexes [7]. Phenolic acids are among the most antioxidant metabolites of cereal crops [8]. The antioxidant properties of phenolic compounds are related to their ability to scavenge free radicals, disrupt radical chain reactions, and chelation of metals. Due to a variety of simultaneous reactions between antioxidants and free radicals, assays based on the mechanism of hydrogen atom transfer (HAT) and single electron transfer (SET) are used to determine antioxidant activity [9]. Due to the simplicity and low cost of the analysis, the ABTS (2,2-azinobis (3-ethylbenzothiazoline-6-sulfonic acid)) assay (based on the HAT mechanism) is employed more often than the DPPH (2,2-diphenyl-1-picrylhydrazyl) assay and FRAP (ferric reducing antioxidant power) assay (both based on the SET mechanism). In addition, the ABTS assay is of particular interest regarding its use for measuring the activity in plant extracts as the absorbance of the wavelength at 734 nm, which is applied for this measurement, eliminates color interference [10]. However, these three tests have shown different results for individual crop species. Yu et al. [11] displayed correlations of antioxidant activity with FRAP and DPPH tests used for wheat samples, whereas Awika et al. [9] observed a high correlation between ABTS, DPPH, and FRAP results among sorghum and its products. Therefore, to show a relationship between the structure of polyphenols from a newly tested waste material and their antioxidant activity, it is advisable to conduct several types of assays to determine the antioxidant activity of the recovered bioactive compounds.

The efficiency of polyphenol recovery depends on the solvent type and the extraction method, which should not chemically modify the extracted compounds. Typically, water or aqueous mixtures of ethanol and methanol in addition to acetone are used to extract antioxidant compounds [12]. Acetone is a slightly toxic solvent that is widely employed in the production of drugs, cosmetics, and in biomedicine. Khan et al. [13] showed that acetone extracts from the *Salvia moorcroftiana* plant exhibit antifungal and antibacterial activity against animal and plant pathogens. Sasmal et al. [14] determined the antipyretic effect of acetone extract from seeds of *Saraca asoca* (Roxb.) in rats. However, to maximize the polyphenol yield, the process parameters should be optimized for the extraction of these compounds from particular waste materials.

The efficiency of extraction of bioactive compounds from biomass can be improved by using physical cell-disruption techniques, such as ultrasound-assisted extraction (UAE) or microwave-assisted extraction (MAE) [4]. For example, optimized MAE can yield up to five times more ferulic acid than conventional solid–liquid extraction techniques [15]. UAE is very efficient, providing, in only an hour, extraction yields more than 6–35% higher than those of conventional extraction methods that often take up to 12 h [12]. Both MAE, based on rapid heating of the solvent by electromagnetic energy (causing molecular movement through ionic conductivity and dipole rotation), and UAE, based on acoustic cavitation, increase the penetration of the solvent into the substrate, improving the mass transfer rate. MAE and UAE offer the following advantages over conventional methods: they can be operated in the absence of light and oxygen; they reduce the consumption of organic solvents; and they can shorten the extraction time [4]. In general, the higher the dielectric constant or the more polar the solvent, the greater its ability to absorb microwaves, thus improving the mass transfer rate, which increases the solvation of the extracted compounds. However, this does not guarantee better extraction results, especially in the case of thermolabile compounds such as polyphenols [15]. Thus, due to the different chemical properties of compounds, it is necessary to optimize MAE and UAE conditions for every solvent and every type of extracted compound. Additionally, the content of polyphenols varies in different types of biomass [16]. For example, Nayak et al. [17] compared MAE and UAE with 51% acetone for the recovery of polyphenols from *Citrus sinensis* peels. They recovered polyphenols of 12.09 mg gallic acid equivalent (GAE)/g with the activity of 337.162 mg Trolox equivalent (TE)/g with MAE and 10.35 mg GAE/g and with 433.084 mg TE/g with UAE. Cherif et al. [18] recovered 94.62 mg ferulic acid equivalent/g from wheat waste with UAE using deep eutectic solvents. The extraction of phenolic compounds from banana peels with MAE using boiling deionized water as a solvent resulted in the content of the phenolic compounds, FRAP, and DPPH antioxidant properties of 53.76 mg GAE/g, 95.52 mg TE/g, and 95.29 mg TE/g, respectively [19]. Thus, because of these very divergent results, there is a need to optimize the conditions of MAE- and UAE-assisted extraction of polyphenols from distillery stillage with a particular solvent.

Several studies reported that the extraction methods and properties of the recovered compounds strongly depend on their structural features and the composition of the matrix, the type of compounds, and the strength of their association with the matrix [16]. Due to their bifunctional nature, phenolic acids can form both ester and glycosidic bonds through reactions involving their carboxyl and hydroxyl groups, respectively, which allow these compounds to form bonds with the cell wall [20]. Phenolic compounds also bind with sugars, fatty acids, and proteins. However, in an acidic environment, esters and glycosidic bonds may be hydrolyzed, and microwaves and ultrasounds may affect the release of phenolic acids from the matrix, increasing the amount of free phenolic acids. Although the hydrolysis conditions significantly affect the overall yield and profile of the phenolic acids, p-coumaric, caffeic, and ferulic acids are degraded under hot acidic conditions, which may reduce the antioxidant activity of the extract [21]. Therefore, to maximize the polyphenol extraction yield and to completely determine the quantity and quality of phenolic acids that maintain their antioxidant activity, it is important to optimize hydrolysis conditions and to consider bound phenolic compounds as most of the phenolic compounds in plant materials are present in this form [21].

The optimization of polyphenol recovery from distillery stillage with UAE and MAE and determination of the antioxidant activity of the extracts represents a novel approach to the utilization of cereal processing by-products generated in alcohol production. Therefore, the main objectives of this study were to investigate polyphenol recovery from distillery stillage with the use of acetone as a solvent to determine how UAE and MAE conditions (extraction time, solvent concentration, and solid-to-solvent ratio) affect (i) the yield of total polyphenols, (ii) the content of flavonoids in the extracts, (iii) the type and content of phenolic acids in the extracts, (iv) kinetic parameters of extraction, and (v) the antioxidant activity of the extracts.

## 2. Materials and Methods

### 2.1. Sample Preparation

Distillery stillage was collected after the production of concentrated crude ethyl alcohol from cereals, mainly wheat and rye, in a distillery located in northeast Poland. The distillery stillage samples contained the following characteristics: 79.9 ± 3.0 g Total Solids/kg fresh weight, 76.2 ± 2.4 g Volatile Solids/kg fresh weight, 47,000 ± 5300 mg COD/L, 4345 ± 5 mg N_tot_/L, 280 ± 2 mg P_tot_/L, 789 ± 3 mg CH_3_COOH/L, alkalinity of 182.8 ± 1.4 meq/L, and a pH of 4.52. After transportation to the laboratory, the stillage was frozen and then freeze-dried (STERIS Lyovac GT2 freeze dryer with a Leybold Trivac vacuum pump, Hamburg, Germany). The freeze-dried samples were stored in tightly closed containers until extractions were performed.

### 2.2. Extraction Procedure

For polyphenol extraction, 1 g of the freeze-dried sample of distillery stillage was taken to each extraction vessel. UAE and MAE were performed with aqueous solutions of acetone at concentrations of 40%, 60%, 80%, and 100%. For the extraction, freeze-dried samples of the stillage were mixed with acetone at ratios of 1:15 (*w*:*v*) and 1:30 (*w*:*v*). UAE was carried out at a frequency of 25 kHz in an ultrasonic bath (InterSonic IS-5.5, Olsztyn, Poland) in a continuous mode. Water remained circulating in the bath to maintain a stable temperature and to avoid overheating the sample as an effect of cavitation. The extraction times were 3, 5, 10, 15, and 20 min. MAE was conducted in a microwave oven (MARSXpress 240/50, CEM, Matthews, NC, USA) in TFM extraction vessels for 1, 3, 5, 8, and 10 min at 400 W and 50 °C. The UAE and MAE extracts were centrifuged (Centrifuge MPW-380, Warszawa, Poland) for 10 min at 10,000 rpm. In the supernatants (final extracts) pH (pH meter, HANNA Instruments HI 221, Cluji-Napoca, Romania), and surface tension (ST) (K100C tensiometer, Hamburg, Germany) were measured at room temperature.

### 2.3. Determination of Total Polyphenol Content (TPC) and Total Flavonoid Content (TFC)

TPC was measured according to the method described by Singletion et al. [22] with modifications. Briefly, 0.25 mL of each extract was mixed with 0.25 mL of Folin–Ciocalteu reagent (F-C) (Sigma-Aldrich, Saint Louis, MO, USA) and 0.5 mL of 14% Na_2_CO_3_. Then, the mixture was incubated for 30 min in the dark at room temperature. TFC was determined according to Quettier-Deleu et al. [23] with modifications. Briefly, 0.5 mL of the extract was mixed with 0.5 mL of 2% AlCl_3_ and 1 mL of 5% NaNO_2_ and incubated for 40 min in the dark at room temperature. TPC and TFC were measured with a UV-Vis spectrophotometer (Varian Cary 50 Scan UV-Visible, Belrose, Australia) at 760 nm and 415 nm, respectively. TPC was expressed as milligrams of gallic acid equivalent per gram dry mass (mg GAE/g DM) and TFC as milligrams of quercetin equivalent per gram dry mass (mg QUE/g DM). Gallic acid and quercetin for curve calibration was sourced from Sigma-Aldrich (Saint Louis, MO, USA).

### 2.4. Determination of Phenolic Acid Content

The collected final extracts from UAE and MAE were evaporated to dryness (Vacuum Rotavapor R-210, BUCHI, Flawil, Switzerland) at 50 °C. Then, 20 mL of distilled water acidified to pH 2 with 6M HCl was added to the dry extracts to dissolve the precipitate formed by evaporating the solvent. Free phenolic acids were extracted from the aqueous solution four times using diethyl ether. The diethyl ether extracts were dried on a rotary vacuum evaporator and redissolved in 1 mL of methanol. The samples thus prepared were used for chromatographic analysis (HPLC).

For recovery of bound phenolic acids, hydrolysis was performed. Distillery stillage (1 g) was mixed with 20 mL of 2M NaOH for 4 h. Then, the mixture was acidified to pH 2 with 6M HCl and centrifuged (Centrifuge MPW-380, Warszawa, Poland) for 10 min at 10,000 rpm. The supernatants were extracted four times with 20 mL of diethyl ether. The mixture of phenolic acids and diethyl ether was evaporated to dryness using a rotary vacuum evaporator. The dry solid was dissolved in 1 mL of methanol (purity: 99.9%, Sigma-Aldrich, SL, USA) and then the phenolic acids were identified by HPLC.

Free and bound phenolic acids were identified chromatographically, according to the method of Chiremba et al. [24] with modifications. The compounds were separated by an HPLC device (Varian, Belrose, Australia) equipped with a UV-Vis detector (Varian ProStar 325, Belrose, Australia) and a Supelcosil C18 column (150 mm × 4.6 mm, 5 µm) (Sigma-Aldrich, Saint Louis, MO, USA). The mobile phase of the elution comprised acetonitrile and formic acid at a ratio of 99.85:0.15 (*v*:*v*) (eluent A) and water and formic acid at a ratio of 99.85:0.15 (*v*:*v*) (eluent B). The purity of the acetonitrile measured 99.9%, formic acid was 98–100% and water PLUS was sustainable for HPLC (Sigma-Aldrich, Saint Louis, MO, USA). The separation time was 42 min at a flow rate of 1 mL/min and a temperature of 35 °C. The analysis used the following gradient elution: 0–18 min, 1–96% B; 18–35 min, 96–82% B; 35–40 min, 82–75% B. P-OH benzoic, vanillic, and syringic acids were detected at 260 nm; p-coumaric, ferulic and sinapic acids, at 320 nm. Identification of compounds was achieved by comparison to the retention time and UV spectra of reference phenolic acids (Sigma-Aldrich, Saint Louis, MO, USA). The purity of the standards measured 95–99%. Due to the lack of reports on the concentration of phenolic acids in by-products from alcohol production from cereals, a wide range of standard solutions was used to render the calibration curves.

### 2.5. Antioxidant Activity

The antioxidant activity of polyphenols in the extracts was measured with ABTS (2,2-azinobis (3-ethylbenzothiazoline-6-sulfonic acid)), DPPH (2,2-diphenyl-1-picrylhydrazyl), and FRAP (ferric reducing antioxidants power) assays.

#### 2.5.1. ABTS Radical Scavenging Assay

The ABTS free radical scavenging assay was performed as described by Re et al. [25] with some modifications. Briefly, 10 mL of ABTS (Sigma-Aldrich, Saint Louis, MO, USA) was mixed with 0.1 mL K_2_S_2_O_8_ and incubated in the dark at room temperature for 16 h. Then, 3 mL of the solution was mixed with 40 µL of the extract and incubated in the dark at room temperature for 6 min. The absorbance at 734 nm was measured (Varian Cary 50 Scan UV-Visible Spectrophotometer, Belrose, Australia). The results were expressed as micromoles of Trolox equivalent per gram dry mass (µmol TE/g DM). Trolox was sourced from Sigma-Aldrich (Saint Louis, MO, USA).

#### 2.5.2. DPPH Radical Scavenging Assay

The DPPH free radical scavenging assay was performed according to Moure et al. [26]. Briefly, 2 mL of DPPH solution (Sigma-Aldrich, Saint Louis, MO, USA) was mixed with 0.2 mL of extract, then the mixture was incubated in the dark at room temperature for 16 min. The absorbance at 515 nm was measured (Varian Cary 50 Scan UV-Visible Spectrophotometer, Belrose, Australia). The results were expressed as micromoles of Trolox equivalent per gram dry mass (µmol TE/g DM).

#### 2.5.3. FRAP Assay

The reducing capacity of the extracts was determined by the FRAP assay, according to Benzie and Strain [27]. FRAP reagent (Sigma-Aldrich, Saint Louis, MO, USA) and extract were incubated for 10 min at 37 °C. The absorbance at 593 nm was measured (Varian Cary 50 Scan UV-Visible Spectrophotometer, Belrose, Australia). The ability of the sample to reduce iron ions was calculated based on a calibration curve made by preparing an FeSO_4_ solution. The results were expressed as micromoles of FeSO_4_ equivalent per gram dry mass (μmol FeSO_4_/g DM).

### 2.6. Calculations and Statistical Analysis

All extractions were performed in duplicate, and analyses were performed in triplicate. Spearman’s rank correlation coefficient (r_s_) was used to quantify the relationships between polyphenol content, antioxidant activities, and extraction parameters of UAE and MAE. The correlation matrix was visualized using a correlogram with RStudio Version 1.2.1335 using the “corrplot” package. For the statistical analysis, STATISTICA 13.1 (StatSoft) was used, and *p* ≤ 0.05 was defined as significant.

To describe the effect of operating parameters on the extraction kinetics, second-order kinetics was used [28], which can be expressed as in Equations (1) and (2):

(1) $$C_{t}=\frac{k\cdot t\cdot C_{e}^{2}}{1+k\cdot t\cdot C_{e}}$$

(2) $$r=k\cdot C_{e}^{2}$$

where: *C_t_*–concentration of phenolic acids (µg/g DM) in the extract at an extraction time *t* (min), *k*–the rate constant for extraction (g DM/(µg·min)), *C_e_*–equilibrium concentration of the compound (µg/g DM) in the extract, *r*–the rate of extraction (µg/(g DM·min)).

## 3. Results and Discussion

### 3.1. Polyphenol Extraction Assisted by Microwaves and Ultrasounds

Extraction of bioactive compounds from waste is a complex process involving various steps such as penetration of solvent molecules, the release of matrix-related compounds, and the dissolution of compounds in the solvent that is based on their affinity to the solvents. Therefore, in this study, optimization of recovery of polyphenols from distillery stillage included investigation of the solid-to-solvent ratio, the concentration of solvent, and the duration of MAE and UAE.

**Figure 1:**
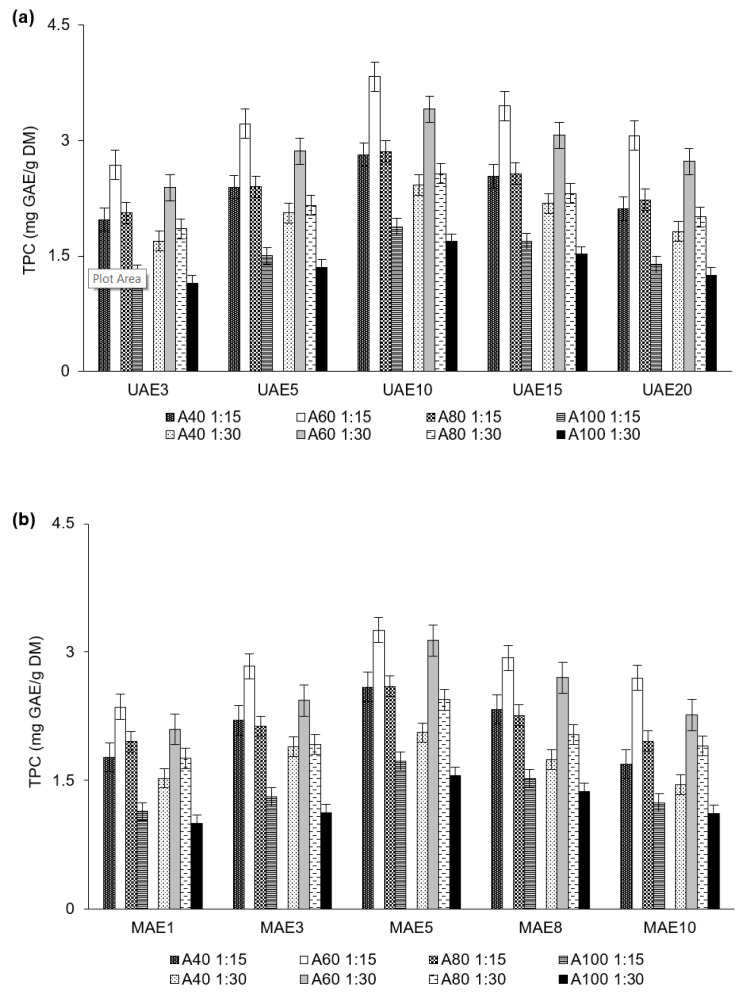
TPC that was extracted from distillery stillage with UAE (**a**) and MAE (**b**). In the abbreviations used to refer to the series, the values after UAE and MAE indicate the extraction time; the values after A (acetone) show the solvent concentration; and finally, the solid-to-solvent ratio is given.

The authors’ previous study on the use of ethanol and methanol for extracting polyphenols from distillery stillage provided the observation that UAE and MAE produced about 2-times higher polyphenol yield with over 9-times shorter extraction times than conventional solid–liquid extraction. Therefore, in this study, the authors focused on the UAE and MAE of polyphenols from distillery stillage. When using acetone as a solvent, the compound yield extracted by UAE was close to that of MAE, but higher TPC and TFC values were still obtained from UAE than from MAE. The TPC in the extracts ranged from 1.15 ± 0.23 mg GAE/g DM to 3.83 ± 0.61 mg GAE/g DM with UAE (Figure 1a) and from 1.00 ± 0.12 mg GAE/g DM to 3.26 ± 0.45 mg GAE/g DM with MAE (Figure 1b). The TFC values ranged from 0.24 ± 0.18 mg QUE/g DM to 0.72 ± 0.58 mg QUE/g DM with UAE and from 0.21 ± 0.09 mg QUE/g DM to 0.61 ± 0.17 mg QUE/g DM with MAE (Table 1). The microwave energy and acoustic cavitation represent the main factors that affect the efficiency of phenolic compound recovery in MAE and UAE, respectively. During UAE, acoustic cavitation causes cavitation bubbles to accummulate at sufficiently high pressures and then to collapse or implode, releasing large amounts of energy. This simultaneously causes high shear forces and turbulence around the cavitation bubbles. Electromagnetic energy in MAE also disturbs the structure of the distillery stillage particles, which leads to the release of compounds from the material matrix. However, excessively high microwave energy in MAE and over-pressurization from ultrasound cavitation in UAE may contribute to overheating the sample. Although an increase in temperature may result in an increase in solubility, diffusion rate and mass transfer of polyphenols, due to a decrease in viscosity of extraction solvent, polyphenols are sensitive to high temperatures [29]. To avoid degradation of these bioactive compounds in the present study, UAE was conducted at room temperature and MAE was conducted at 50 °C. On the other hand, the recovery of phenolic compounds from plant materials that are more closely bound to the cell wall [17,19,29], requires higher inputs of energy, temperature, and pressure for their release. Therefore, the first step in the recovery of phenolic compounds is to identify the most effective MAE and UAE conditions. In our preliminary study, the most effective level of the microwave was identified as 400 W, and the most effective amplitude of ultrasound treatment was found to be 100%. Compared to conventional heating processes, microwave heating involves a rapid increase in volume temperature without surface overheating, better process control, and higher energy efficiency, with fewer adverse effects on antioxidant activity and bioactive content [17]. Additionally, microwaves induce translational and rotational molecular movement by changing the structure of enzymes without directly breaking the covalent bonds between the polar groups in the enzyme [30]. Marszałek et al. [31] reported that polyphenol oxidase activity in strawberries was reduced by 80% during microwave heating (between 300 and 900 W). Therefore, microwaves may inhibit the enzymatic degradation of polyphenols and ensure their higher stability.

#### 3.1.1. Effect of Extraction Time on Polyphenol Recovery

The duration of extraction plays a key role in the recovery of phenolic compounds as it affects the stability of the bioactive compounds since UAE and MAE generate heat, and, in addition, obtaining a high yield over a shorter time period is much more productive. When the time of UAE was increased from 3 to 10 min, the amount of phenolic compounds that were extracted increased by about 43%, but further extension of this time decreased the recovery (Figure 1a, Table 1). At the longest duration of 20 min, the content of extracted compounds was similar to that obtained at 3 min. Regarding MAE, increasing the extraction time from 1 to 5 min increased the content of recovered polyphenols by about 38% (Figure 1b, Table 1). Further increasing the duration of MAE to 10 min gradually decreased the extraction yield; the content of polyphenols extracted with 10-min MAE was similar to that with 1-min MAE. These findings showed that UAE and MAE had finished by 10 min and 5 min, respectively. The most efficient time of UAE resulted in 17% higher polyphenol yield than the most efficient time in MAE since the sample disruption by microwaves could have been less than that by ultrasonic treatment, as reported by SEM images of olive pomace extracted with these methods [28]. In the present study, after 10 and 5 min of UAE and MAE, the TPC, TFC, and phenolic acid content were negatively correlated with the time of extraction (r_s_ = (−0.31) − (−0.44); *p* ≤ 0.05) (Figure S2). Although MAE and UAE partially break the polyphenol network into low molecular weight polyphenols, allowing intracellular bioactive compounds to be released in a short time, polyphenols can degrade if they are exposed to microwaves and ultrasound for excessive time periods. In the present study, the decrease in polyphenol yield with time period durations of UAE above 10 min and MAE above 5 min could be due to the production of free hydroxyl radicals, which allows highly active substances to break the chemical structure of polyphenols, especially when water content is high, and due to temperature increase [28]. Acoustic cavitation or high temperatures promote reactions of oxidation, polymerization, and decomposition, thus generating other products. Therefore, for example, a study by Qiao et al. [32] showed that four phenolic acids (protocatechuic acid, p-OH benzoic acid, vanillic acid, and p-coumaric acid) were stable, while three phenolic acids (caffeic acid, sinapic acid, and ferulic acid) were degraded by ultrasound. Regarding temperature, it should not have caused decomposition of polyphenols in the present study, since extractions were conducted at a temperature that did not exceed a safe value for thermolabile polyphenols [29].

#### 3.1.2. Effect of Solid-to-Solvent Ratio on Polyphenol Recovery

The efficiency of recovering phenolic compounds from distillery stillage was about 17% higher (UAE) or about 14% higher (MAE) at a solid-to-acetone ratio of 1:15 than at 1:30 (*w*:*v*) (Figure 1a,b, Table 1). This can be explained by the fact that the strength of the solvent was so high that a smaller amount of solvent was sufficient for recovering phenolic compounds, while a larger amount diluted the sample. Additionally, it should be noted that, after establishing equilibrium in the phenolic compound recovery reaction, there is a steady state where an increase in the amount of the solvent does not affect recovery efficiency [33]. Therefore, in the present study, increasing the solvent volume did not improve the efficiency of phenolic compound extraction. A similar result during extraction of bioactive compounds from olive leaves was explained by differences in concentrations of these compounds between the interior and exterior of the olive leaf cell, which affected the mass transfer in extraction [34]. The fact that a lower acetone-to-solid ratio favored extraction yield is beneficial concerning both the extraction costs and the concentration and purification of extracts. However, to interpret the effect of solvent quantity on polyphenol recovery more comprehensively, the affinity of the solute for the solvent was determined, including behaviors attributed to hydrogen bonding and polar interactions (Section 3.1.3).

#### 3.1.3. Effect of Solvent Concentration on Polyphenol Recovery

TPC and TFC decreased as acetone concentrations were changed in the following order: 60% > 80% > 40% > 100% (Figure 1a,b, Table 1). Using 60% acetone produced about 2-times higher polyphenol yield than using 100% acetone. The TPC, TFC, and phenolic acid content were strongly negatively correlated with the solvent concentration (r_s_ = (−0.90) − (−0.97); *p* ≤ 0.05) (Figure S2). Extraction procedures, including solvent characteristics, strongly affect the recovery of phenolic compounds, which dissolve better in polar organic solvents than in non-polar ones, due to the presence of hydroxyl groups in the polar solvents [28]. The addition of water to the solvent reduces the dielectric constant, increasing solvent diffusion and desorption of the solute from the sample. Therefore, in the present study, aqueous mixtures of acetone were more effective in recovering polyphenols than pure acetone. Similarly, 60% acetone proved the best solvent concentration for the recovery of phenolic compounds from brewers’ spent grain (4.00 mg GAE/g DM) [35] and *Salsola tomentosa* (TPC of 20.37 mg GAE/g and TFC of 2.08 mg QUE/g) [36]. All these observations show that approaching the polarity of the solvent to the polarity of solutes, thus increasing the surface area of the solute–solvent contact, improves extraction yield [28]. This confirms the necessity of optimizing the solvent concentration for extracting particular compounds from plant materials.

To better explain the relationship between acetone concentration and polyphenol extraction yield, the Hansen Solubility Parameter (HSP) was employed to assess the solubility of phenolic compounds in acetone. The individual six phenolic acids that were detected in the extracts were selected for these calculations. The theory of the HSP is based on the cohesion energy between molecules, which is described in terms of dispersion forces (D), polarity (P), and the energy of intermolecular hydrogen bonds (H). Respective solubility parameters (δ_D_, δ_P_, and δ_H_) were calculated based on molar volume (V_m_) and group contributions to individual solubility parameters (F_D,i_, F_P,i_ and F_H,i_), based on Hansen [37]. The affinity of phenolic acids for acetone and water was assessed based on the values of the total solubility parameters (δ_T_) (Equations (3)–(6)) (Table 2).

(3) $$\delta_{D}=\frac{\sum F_{D,i}}{V_{m}}$$

(4) $$\delta_{P}=\frac{\sqrt{\sum F_{P,i}^{2}}}{V_{m}}$$

(5) $$\delta_{H}=\frac{\sqrt{\sum F_{H,i}}}{V_{m}}$$

(6) $$\delta_{T}^{2}=\delta_{D}^{2}+\delta_{P}^{2}+\delta_{H}^{2}$$

The affinity between the molecules is greater the smaller the difference between the δ_T_ values for each one [37]. The dispersion parameter (δ_D_) was similar for all tested substances, meaning it did not bear a significant effect on the miscibility and solubility of these components. From Table 2 it can be concluded that the polarity (δ_P_) and the hydrogen bond energy (δ_H_) exhibited the largest effect on the affinity of the solutes for the solvents. The greatest differences were observed in the case of the energy of hydrogen bonds for water and phenolic acids, which suggests that, under normal conditions, water is the least compatible of the tested solvents for phenolic acid extraction. Therefore, in this study, mixtures of water and acetone at different concentrations were used as the solvent to determine the optimal solvent composition for the recovery of phenolic compounds. Therefore, it was necessary to determine the HSP values for each composition of the mixture.

In the case of multi-component solvents, the HSP of the solvent (δ_mix_) is calculated from the volume fraction of the individual components in the solvent mixture (φ_i_) and their contributions to the HSP of the mixture (δ_i_). Finally, to determine the affinity of multi-component solvents (aqueous acetone solutions) for specific phenolic acids, the distance between the points representing the solute (δ_D,A_, δ_P,A_, δ_H,A_) and the solvent (δ_D,B_, δ_P,B_, δ_H,B_) was calculated, which represents the Hansen solubility parameter distance (R) (Equations (7) and (8)).

(7) $$\delta_{\mathrm{mix}}=\underset{i}{\sum }\varphi_{i}\delta_{i}$$

(8) $$R=\sqrt{4{\left(\delta_{D,A}-\delta_{D,B}\right)}^{2}+{\left(\delta_{P,A}-\delta_{P,B}\right)}^{2}+{\left(\delta_{H,A}-\delta_{H,B}\right)}^{2}}$$

**Figure 2:**
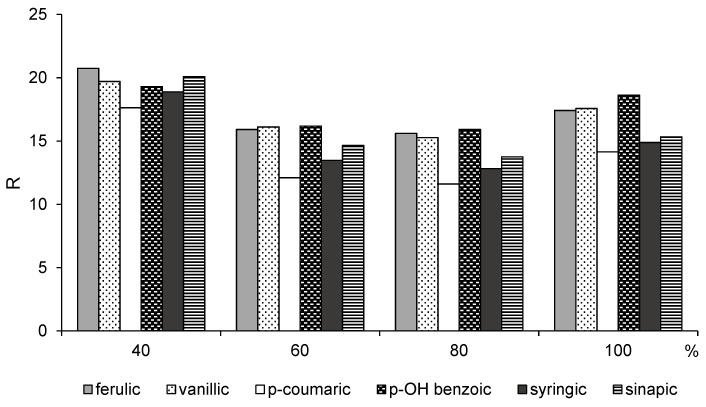
Hansen solubility parameter distances (R) between phenolic acids and 40%, 60%, 80%, and 100% acetone.

Optimal selection of the components of the solvent mixture can improve the efficiency of phenolic acid recovery. Thus, the observed differences in the R parameter were dependent on the concentration of acetone in the solvent mixture (Figure 2).

The R values for phenolic acids in aqueous acetone solutions revealed a non-monotonic trend with the minimum values of this parameter (12.11–16.18 and 11.61–15.92, respectively) at 60% and 80% (*v*:*v*) acetone (Figure 2). Low R values indicate high solubility as the interaction forces between the molecules of all components are similar. It was observed that acetone–water mixtures were the most effective solvents for extraction of the mixture of phenolic acids recovered from stillage as the phenolic acids that were extracted differ only slightly in their structure. This is because adding water to acetone increases the entropy of the mixture, and thus the structure of the solvent mixture becomes less ordered, which favors the interaction of phenolic acids with the solvent mixture [35]. Therefore, the HSP provides a simple method of predicting the solvent composition that will most efficiently recover bioactive compounds.

However, whereas the HSP analysis predicted that 60% and 80% aqueous solutions of acetone would recover phenolic acids with equal efficiency (Figure 2), the efficiency of TPC recovery was significantly higher with the 60% solution (Figure 1). This discrepancy resulted from the fact that the mixture of phenolic acids in the real sample created more complex natural products. The affinity of these compounds for solvents at different concentrations and the resulting selectivity of extraction are determined by various interactions between substrate components and the bioactive compounds, which is influenced by their chemical structure, overall composition, and the type of biomass from which the bioactive compounds are recovered [38].

#### 3.1.4. pH and ST of the Extracts

The pH and ST of the extracts affect the stability and solubility of the bioactive compounds, which in turn affect the efficiency of extraction and the antioxidant activity of the extracts. Therefore, these two factors were monitored when optimizing the recovery of polyphenols. The pH of the UAE extracts ranged from 3.30 to 5.11, and that of the MAE extracts, from 3.36 to 4.98. Low pH significantly improves the recovery of phenolic compounds. Friedman and Jurgens [39] observed that in extracts with a pH of 1, the recovery of phenols from grape skins was three times higher than in those with a pH of 6 or 8. This is because phenolic compounds are more stable under acidic conditions than under neutral conditions. In addition, under acidic conditions, phenolic compounds are released from the cell wall, resulting in high extraction efficiency [40].

In the present study, the ST of the extracts ranged from 22.63 to 29.90 mN/m (UAE) and from 22.57 to 29.68 mN/m (MAE). The ST values in the UAE and MAE extracts were lowest when using 60% acetone. This allowed easier penetration of acetone into the distillery stillage matrix, which increased the contact surface between the matrix and the solvent [34]. Therefore, the acidic pH and low ST of the extracts favored the transport of phenolic compounds into the solvent.

### 3.2. Phenolic Acids

#### 3.2.1. Content of Phenolic Acids in the Extracts

All extracts displayed similar phenolic acid profiles, although the concentrations of free and bound phenolic acids varied. The average total content of phenolic acids (after hydrolysis) was 2.34 ± 0.23 µg/g DM. The concentrations of free phenolic acids ranged from 0.79 to 2.12 µg/g DM and from 0.59 to 1.78 µg/g DM with UAE, and from 0.72 to 1.89 µg/g DM and from 0.53 to 1.58 µg/g DM with MAE, at solid-to-solvent ratios of 1:15 and 1:30 (*w*:*v*), respectively. In the most efficient extraction variants of UAE and MAE, free phenolic acids accounted for 81–91% of the total phenolic acid content, respectively. The high level of free phenolic acids in the extracts resulted from the fact that distillery stillage is generated during the anaerobic processing of plant materials. In general, fermentation processes release bound compounds and increase their extractability from plant-origin materials [40].

**Figure 3:**
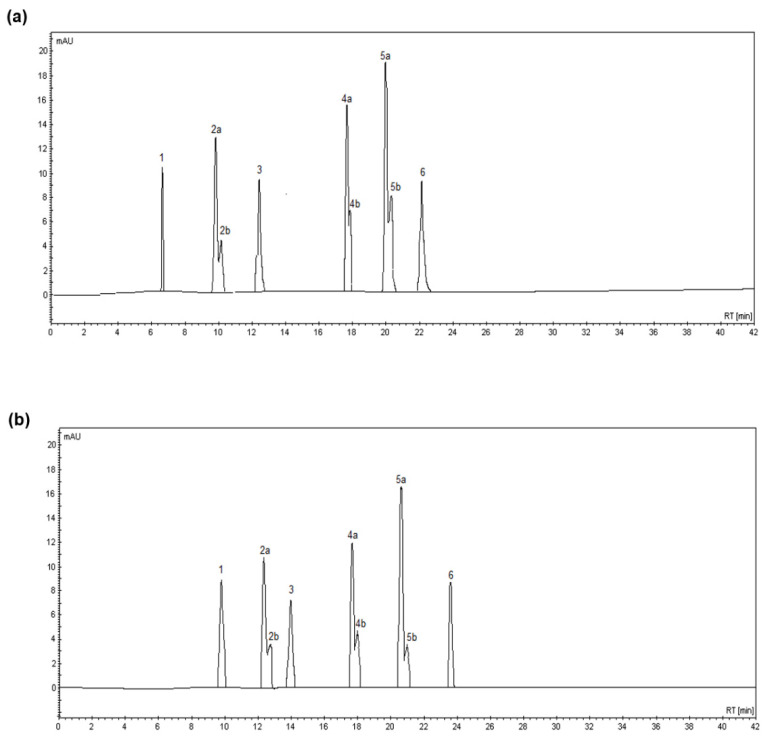
Chromatograms of phenolic acids in extracts from UAE (**a**) and MAE (**b**) with 60% acetone at a solid-to-solvent ratio of 1:15 (*w*:*v*). Peaks: (**1**) p-OH benzoic acid; (**2a**) vanillic acid; (**2b**) oxidized vanillic acid; (**3**) syringic acid; (**4a**) p-coumaric acid; (**4b**) oxidized p-coumaric acid; (**5a**) ferulic acid; (**5b**) oxidized ferulic acid; and (**6**) sinapic acid.

In all extracts, the phenolic acid fraction consisted of six acids: p-OH benzoic acid, vanillic acid, syringic acid, p-coumaric acid, ferulic acid, and sinapic acid (Figure 3). Vanillic, p-coumaric, and ferulic acids were detected with two characteristic peaks; the left peak indicated the unoxidized species (a) while the right peak indicated the more polar oxidized species (b). All three compounds contain a double bond in the carboxylic acid hydrocarbon chain, which is likely to be susceptible to oxidation [41]. Although the retention times of a particular phenolic acid differed slightly depending on whether it was in a UAE or an MAE extract (Figure 3), this did not prevent the identification of the acids. In addition, each peak was separated from the others, indicating that the extraction procedure was performed correctly and that one acid was not transformed into another one.

**Figure 4:**
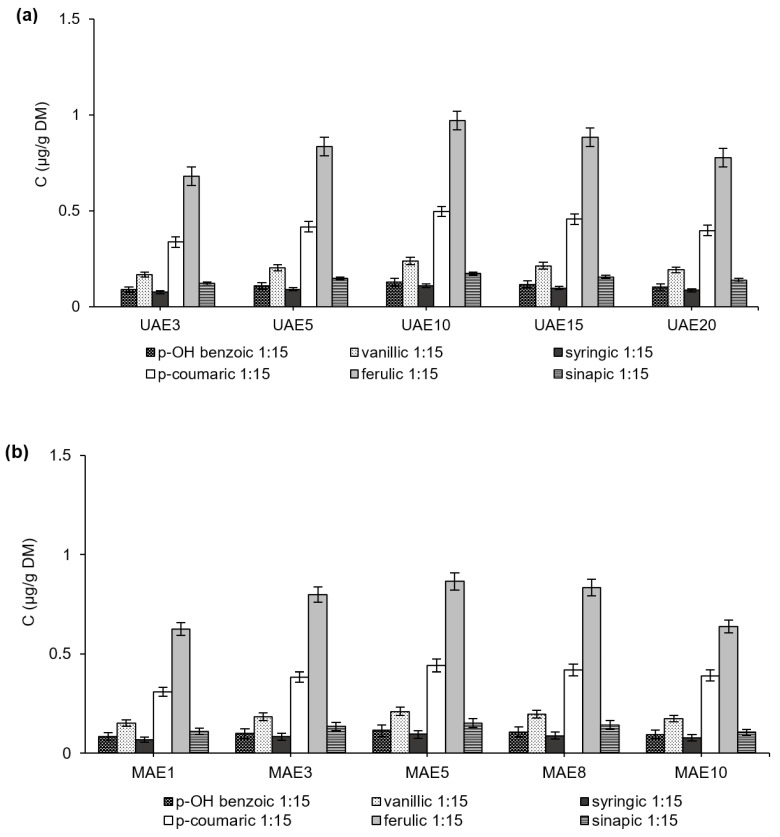
Concentrations of individual phenolic acids in extracts from UAE (**a**) and MAE (**b**) with 60% acetone. In the abbreviations used to refer to the series, the values after UAE and MAE indicate the extraction time.

The recovery of free phenolic acids was highest when using 60% acetone during 10-min UAE at the solid-to-solvent ratio of 1:15 (*w*:*v*), with total values of 2.12 µg/g DM (Figure 4). In the case of MAE, the concentrations of phenolic acids were lower, amounting to 1.88 µg/g DM with 5-min extraction with 60% acetone at a solid-to-solvent ratio of 1:15 (*w*:*v*). The concentrations of individual free phenolic acids obtained in other variants of UAE and MAE are shown in Figure S1.

In the phenolic acid fraction of the extracts from distillery stillage, ferulic acid and p-coumaric acid predominated, with respective shares ranging from 40 to 46% and from 19 to 23%, respectively, depending on the extraction conditions (Figure 4). These acids were followed by vanillic and sinapic acids, with shares of 12–15% and 7–10%, respectively, and then by p-OH benzoic and syringic acids, which together accounted for about 4–6% of the total. Skrajda-Brdak et al. [42] discovered similar proportions of phenolic acids in wheat, rye, and oat grain. The composition and proportion between phenolic acids extracted with a particular solvent are related to the chemical structure of phenolic acids. Phenolic acids are classified as hydroxybenzoic acids or hydroxycinnamic acids [21]. Hydroxybenzoic acids include p-OH benzoic, vanillic, and syringic acids, while hydroxycinnamic acids include p-coumaric, ferulic, and sinapic acids; all these acids exist in the form of esters and glycosides. In the present study, the finding of predomination of ferulic and p-coumaric acids was attributed to the similarity in their chemical structures, namely the presence of a –CH=CH-COOH group. The C=C double bond participates in the stabilization of ferulic and p-coumaric acids, hence these acids were resistant to degradation [43]. The highest concentrations of ferulic and p-coumaric acids may be due to their location in the cell wall in the cereals from which the alcohol was produced. These acids appear both in arabinose esterified bonds in hemicelluloses and etherified lignin bonds (while other phenolic acids are mostly esterified to lignin) [21]. This explains why ferulic and p-coumaric acids were released rather than degraded during alcoholic fermentation as might have been the case with other phenolic acids. Additionally, in other studies, it was noticed that vanillic acid may transform into ferulic acid due to physical and chemical interactions [20], which may result in a higher concentration of ferulic acid in extracts in the present study.

The phenolic acids that were extracted from the distillery stillage in the present study exhibit valuable properties. Ferulic acid protects against coronary artery disease, lowers serum and liver cholesterol levels, and increases sperm viability [44]. P-coumaric acid contains chemoprotective and antioxidant properties [45]. Vanillic acid is the major metabolic product of vanillin aldehyde, which exhibits antimicrobial, antimutagenic, and anti-carcinogenic effects [46]. Sinapic acid acts as a powerful oxidant scavenger, thus protecting cellular components [47]. P-OH benzoic acid has antimicrobial activity and is widely used as a preservative in food, cosmetic, and pharmaceutical products [32]. Syringic acid restores insulin sensitivity, increases glucose consumption, and is involved in carbohydrate metabolism [48].

#### 3.2.2. Kinetics of UAE and MAE

The appropriate conditions for the rapid extraction of phenolic acids from distillery stillage were confirmed by the study of extraction kinetics. In Table 3, extraction rates are given, which were calculated for the ranges 0–10 min (UAE) and 0–5 min (MAE). For all experimental conditions, the determination coefficient R^2^ was 0.99, indicating that the equations of the second-order kinetics adhered well to the subsequent experimental results. The content of total phenolic acids in the extracts increased with the maximal rates of 1.14 µg/(g DM·min) (UAE) and 3.93 µg/(g DM·min) (MAE) at a solid-to-solvent ratio of 1:15 (*w*:*v*) with 60% acetone. At each operating condition and for each phenolic acid, the extraction rate in MAE measured about 3 times higher than that of UAE. This may indicate that heat transfer was responsible to a higher extent for the rate of dissolution of solute than the cavitation effect. Moreover, ultrasound waves are considered to affect the extraction process but not diffusion through the solid particles [28]. However, when extracting bioactive compounds from olive pomace, the extraction rates were 1.1, 3.7, and 1.1 times higher in UAE than in MAE, for hydroxytyrosol, maslinic acid, and oleanolic acid, respectively [28]. These contradictory results suggest that different extraction methods should be optimized each time when extracting compounds from different types of waste material.

### 3.3. Antioxidant Activity

To determine the usefulness of recovered phenolic acids, their antioxidant activity was determined. This activity depends on the type of substrate, the recovery procedures, and the cereal growing conditions since the active compounds are usually synthesized in response to stress, such as attack by microorganisms or strong UV radiation [46].

**Figure 5:**
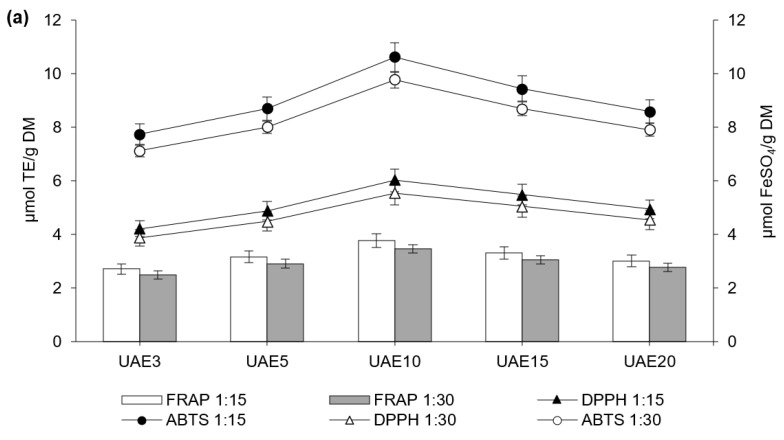

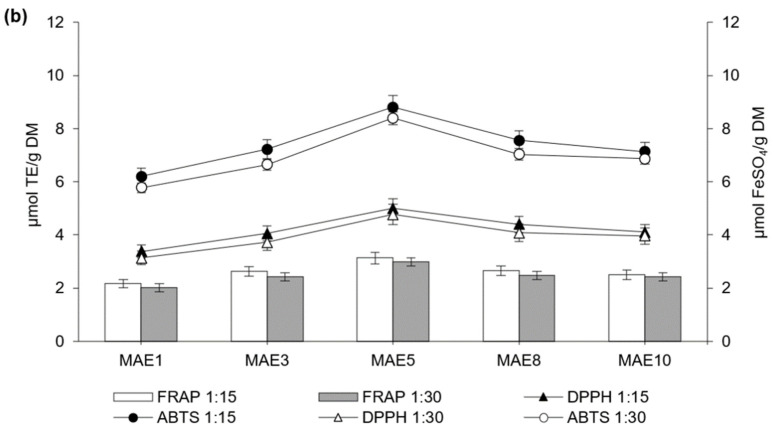
Antioxidant activity of the extracts from UAE (**a**) and MAE (**b**) with 60% acetone. In the abbreviations used to refer to the series, the values after UAE and MAE indicate the extraction time.

In the present study, the antioxidant activity of the extracts from the stillage was assessed employing ABTS, DPPH, and FRAP assays. Figure 5 displays the antioxidant activity of polyphenols extracted with UAE and MAE using 60% acetone. The antioxidant activity of extracts obtained with other variants of UAE and MAE is shown in Table S1.

The antioxidant activities of extracts obtained with 60% acetone were significantly higher than those of the extracts obtained with the other acetone concentrations. These activities correlated positively with the content of phenolic acids (Figure S2); therefore, it can be assumed that these compounds were responsible for the antioxidant properties of the extracts. Since the activity was strongly dependent on the content of phenolic acids, the activity of extracts obtained with 10-min UAE measured the highest, as indicated by ABTS (9.76–10.60 μmol TE/g DM), DPPH (5.54–6.03 μmol TE/g DM), and FRAP (3.47–3.77 μmol FeSO_4_/g DM). Slightly lower values of antioxidant activity were obtained when MAE was used for 5 min, as indicated by ABTS (8.39–8.80 μmol TE/g DM), DPPH (4.77–5.00 μmol TE/g DM), and FRAP (2.98–3.13 μmol FeSO_4_/g DM). Lengthening UAE beyond 10 min and MAE beyond 5 min decreased extract activity due to lower content of phenolic acids in the extracts. This was not entirely consistent with other studies, in which only increases in extraction time above 45 min decreased the antioxidant activity of mung bean extract [29]. Kwaw et al. [30] observed that 15 min of UAE significantly increased (1.0–1.2 times) the phenolic content and antioxidant properties of mulberry juice. A similar trend was observed in the studies by Mehmood et al. [48], where it was noted that the antioxidant activity of compounds recovered from the butterfly pea flower was significantly higher (1.2–1.4 times) after UAE than after conventional recovery methods.

The results of this study revealed that ferulic and p-coumaric acids, as the acids comprising the highest content in 60% acetone extracts, are the components most responsible for the antioxidant activity of the distillery stillage extracts. Kikuzaki et al. [49] and Strazisar et al. [50] also reported strong antioxidant activity of these two acids. This property is attributed to their structural characteristics. The phenolic nucleus and unsaturated side chain in the structure of ferulic and p-coumaric acids can create stabilized phenoxy radicals, which account for their potent antioxidant activity [43]. The bioactive properties are affected by the size and polarity of the molecules, as well as the modification of the polarity of the substituents on the aromatic ring [51]. All acids with hydroxyl groups on the aromatic ring are highly active, therefore hydroxycinnamic acids are more active than hydroxybenzoic acids [52]. In the case of hydroxycinnamic acids, ferulic acid exhibits a higher antioxidant activity than p-coumaric acid. On the other hand, the presence of polar functional groups on an aliphatic side chain often reduces the antioxidant activity of aromatic compounds [51]. According to Wang and Wang [20], the presence of the carboxyl group reduces the activity to a greater extent than the presence of the hydroxyl group. For this reason, despite the similar structure, syringic acid is less active than ferulic acid, p-coumaric acid, and sinapic acid. The properties of sinapic acid derive from its carboxyl substituent and three other substituents. A similar effect can be observed when comparing the activity of vanillic and p-coumaric acids, which have similar molecular weights. However, p-coumaric acid is more biologically active since it has fewer substituents and a longer side chain than vanillic acid. However, despite knowledge regarding the activity of these individual compounds, real extracts should be investigated in terms of not only their phenolic acid composition but also in terms of their antioxidant activity. Natural extracts may be more beneficial than isolated compounds since the synergistic interactions of compounds can alter the bioactive properties of individual ingredients [50].

In the present study, in all extracts, the ABTS assay indicated significantly higher antioxidant activity than the DPPH assay, which was followed by the FRAP assay. However, values obtained in each of the assays exhibited a strong positive correlation with each other (Figure S2). Similarly, Yu et al. [11] found that the ABTS assay for wheat bread extract showed 10–20 times higher values than that indicated by the DPPH assay. Smuda et al. [8] discovered that the ABTS assay showed higher activity in wheat, rice, and maize by-products than the FRAP assay. One observation of this is that each assay bears a different mechanism, based on HAT or SET. The antioxidant activity of the phenolics is highly dependent on the molecule structure, namely the position and the number of hydroxyl groups. These two assays (ABTS and DPPH) are complementary and provide an abundance of information on the capability of phenolic acids to react with free radicals. The FRAP assay shows a different action since the reaction does not involve free radicals, but relies on the ability of antioxidants to reduce ferric iron (Fe^3+^) to iron (Fe^2+^). As an effect, employing all these assays provides a full picture of antioxidant properties of extracts that contain a mixture of bioactive compounds which can react with free radicals through various mechanisms.

### 3.4. Should UAE or MAE Be Chosen for Polyphenol Recovery?

Although the average content of polyphenols that were recovered from distillery stillage in the present study was lower than that from other substrates like wheat, rice, or maize wastes [44,45], the high availability of distillery stillage, which represents a waste material, throughout the year renders the stillage a promising material for the recovery of polyphenols.

In the present study, UAE allowed for the 17% higher recovery yield of total polyphenols than MAE at the best-operating conditions in both methods. However, these results were obtained at 10-min UAE and 5-min MAE, which resulted from a 3 times higher extraction rate in MAE than in UAE. For these extraction times, UAE required 94 Wh of energy, while MAE required 33 Wh, which in the final economic assessment should bear a significant effect on the selection of the extraction method. Therefore, it can be concluded that although UAE yields higher content of polyphenols, MAE provides a more economical process owing to lower power input due to a shorter extraction time. However, to consider the implementation of these processes in a full industrial scale, other factors describing economic and environmental impact should be evaluated. Xie et al. [28], when comparing kinetic constants and thermodynamic parameters in UAE and MAE for extracting bioactive compounds from olive pomace, found that UAE was the greener and most effective technique of extraction. According to these authors, the E factor, which is defined as the mass ratio of waste to the desired product, was lower in UAE than in MAE by 6% and 13%, at 40 °C and 50 °C, respectively. In addition, energy consumption and corresponding carbon emissions were lower in UAE than in MAE by 14% and 11%, at 40 °C and 50 °C, respectively. When selecting the extraction method, the fact that UAE is employed at low temperatures should be also highlighted, which is particularly important in the extraction of polyphenols, which are temperature sensitive.

When evaluating the economic and environmental effects of extraction, the issue of extract purification should be considered. Effective methods of purifying natural bioactive compounds from plant extracts include solid-phase extractions via adsorption–desorption. This type of purification uses various solid phases such as silica gel, stationary phases for hydrophilic interaction liquid chromatography (HILIC) in reverse phase materials, and ion exchange/polymer resins [53]. The authors obtained the purity of 80–98% by weight after processing extracts of fruit and vegetables with macroporous resins. The isolation and purification processes of commercial phenolic compounds must be cost-effective, scalable, economically feasible, and capable of isolating valuable compounds from plant materials without significant degradation. For this purpose, multi-step processing has been proposed, for example, membrane separation combined with adsorption–desorption. It was observed that crude solvent extracts subjected to adsorption–desorption on macroporous resins maintained high antioxidant activity [53].

Apart from phenolic compounds, bioactive compounds in plant materials include other secondary metabolites such as antibiotics, mycotoxins, alkaloids, food colors, or plant growth factors. Solid–liquid extraction with the use of organic solvents and supported by ultrasound waves and microwaves may be used to recover these compounds [7]. However, bioactive compounds range from very polar to very non-polar. Therefore, when recovering bioactive compounds, the selection of the type and concentration of extraction solvent and extraction conditions plays an important role in the extraction efficiency.

## 4. Conclusions and Future Perspectives

This study showed that distillery stillage provides a valuable source of bioactive polyphenols that can be recovered via UAE and MAE with acetone. A short extraction time (10-min UAE or 5-min MAE) and a 60% acetone solution yielded the highest polyphenol content. Polyphenols were extracted more effectively with UAE, but the balance between the polyphenol yield and the energy consumption of the process proved better with MAE. The antioxidant activity of the extracts strongly correlated with both the total content of polyphenols and the content of individual phenolic acids, indicating that these substances contributed substantially to the bioactive properties of the extracts. A total of six different phenolic acids were recovered in mainly free forms, predominantly ferulic acid and p-coumaric acid. These findings indicate that the phenolic acids recovered from distillery stillage can potentially serve as valuable compounds, thus opening new possibilities for profitable valorization of this waste and an adjustment of the work of distilleries to the assumptions of a circular economy. Future studies should develop the methods of purification and concentration of phenolic compounds to obtain products that will be applicable in the food, cosmetic, and pharmaceutical industries.

## Figures and Tables

**Table 1:** TFC that was extracted from distillery stillage with UAE and MAE. In the abbreviations used to refer to the series, the values after UAE and MAE indicate the extraction time; the values after A (acetone) show the solvent concentration.

	Extraction	TFC (mg QUE/g DM)	Extraction	TFC (mg QUE/g DM)
Solid-to-solvent ratio of 1:15 (*w*:*v*)	UAE3 A40	0.40 ± 0.10	MAE1 A40	0.36 ± 0.44
	UAE3 A60	0.50 ± 0.18	MAE1 A60	0.44 ± 0.36
	UAE3 A80	0.43 ± 0.09	MAE1 A80	0.40 ± 0.38
	UAE3 A100	0.26 ± 0.16	MAE1 A100	0.23 ± 0.27
	UAE5 A40	0.49 ± 0.24	MAE3 A40	0.45 ± 0.31
	UAE5 A60	0.60 ± 0.42	MAE3 A60	0.53 ± 0.56
	UAE5 A80	0.50 ± 0.36	MAE3 A80	0.44 ± 0.41
	UAE5 A100	0.31 ± 0.38	MAE3 A100	0.27 ± 0.22
	UAE10 A40	0.58 ± 0.39	MAE5 A40	0.52 ± 0.58
	UAE10 A60	0.72 ± 0.58	MAE5 A60	0.61 ± 0.17
	UAE10 A80	0.59 ± 0.42	MAE5 A80	0.54 ± 0.55
	UAE10 A100	0.38 ± 0.37	MAE5 A100	0.35 ± 0.43
	UAE15 A40	0.52 ± 0.26	MAE8 A40	0.47 ± 0.33
	UAE15 A60	0.64 ± 0.54	MAE8 A60	0.57 ± 0.41
	UAE15 A80	0.53 ± 0.55	MAE8 A80	0.47 ± 0.22
	UAE15 A100	0.35 ± 0.49	MAE8 A100	0.32 ± 0.18
	UAE20 A40	0.43 ± 0.57	MAE10 A40	0.35 ± 0.19
	UAE20 A60	0.57 ± 0.61	MAE10 A60	0.52 ± 0.59
	UAE20 A80	0.46 ± 0.55	MAE10 A80	0.41 ± 0.27
	UAE20 A100	0.28 ± 0.42	MAE10 A100	0.25 ± 0.12
Solid-to-solvent ratio of 1:30 (*w*:*v*)	UAE3 A40	0.35 ± 0.21	MAE1 A40	0.31 ± 0.16
	UAE3 A60	0.49 ± 0.26	MAE1 A60	0.43 ± 0.38
	UAE3 A80	0.38 ± 0.32	MAE1 A80	0.36 ± 0.41
	UAE3 A100	0.24 ± 0.18	MAE1 A100	0.21 ± 0.09
	UAE5 A40	0.42 ± 0.23	MAE3 A40	0.38 ± 0.27
	UAE5 A60	0.58 ± 0.44	MAE3 A60	0.50 ± 0.42
	UAE5 A80	0.45 ± 0.37	MAE3 A80	0.40 ± 0.39
	UAE5 A100	0.28 ± 0.19	MAE3 A100	0.23 ± 0.27
	UAE10 A40	0.50 ± 0.43	MAE5 A40	0.42 ± 0.34
	UAE10 A60	0.69 ± 0.54	MAE5 A60	0.64 ± 0.59
	UAE10 A80	0.53 ± 0.51	MAE5 A80	0.50 ± 0.39
	UAE10 A100	0.35 ± 0.38	MAE5 A100	0.32 ± 0.26
	UAE15 A40	0.45 ± 0.52	MAE8 A40	0.36 ± 0.14
	UAE15 A60	0.62 ± 0.59	MAE8 A60	0.55 ± 0.48
	UAE15 A80	0.48 ± 0.58	MAE8 A80	0.42 ± 0.44
	UAE15 A100	0.32 ± 0.48	MAE8 A100	0.29 ± 0.22
	UAE20 A40	0.37 ± 0.36	MAE10 A40	0.30 ± 0.19
	UAE20 A60	0.56 ± 0.25	MAE10 A60	0.49 ± 0.34
	UAE20 A80	0.41 ± 0.58	MAE10 A80	0.39 ± 0.26
	UAE20 A100	0.26 ± 0.42	MAE10 A100	0.23 ± 0.29

**Table 2:** Hansen solubility parameters of the solvents and the phenolic acids.

Compound	V_m_ (mL/mol)	δ_D_	δ_P_	δ_H_	δ_T_
water	18.06	15.5	16.0	42.3	47.8
acetone	74.08	15.5	10.4	7.0	19.9
ferulic acid	136.75	22.7	5.7	15.6	28.1
vanillic acid	119.25	22.6	6.5	16.6	28.8
p-coumaric acid	118.10	20.4	5.6	16.0	26.5
p-OH benzoic acid	94.60	22.9	7.0	17.8	29.8
syringic acid	151.28	21.2	5.8	15.4	26.8
sinapic acid	167.32	21.6	5.2	14.7	26.6

**Table 3:** Extraction rates for particular phenolic acids and the total phenolic acids. In the abbreviations used to refer to the series, the values after A show the solvent concentration; and finally, the solid-to-solvent ratio is given.

Type of Extraction	Extraction Rate (µg/(g DM·Min))						
	*p*-OH Benzoic	Vanillic	Syringic	*p*-Coumaric	Ferulic	Sinapic	Total Acids
UAE A40 1:15	0.047	0.085	0.038	0.178	0.348	0.062	0.758
UAE A60 1:15	0.070	0.130	0.058	0.249	0.539	0.095	1.142
UAE A80 1:15	0.054	0.098	0.045	0.206	0.403	0.071	0.878
UAE A100 1:15	0.034	0.062	0.028	0.131	0.255	0.045	0.557
UAE A40 1:30	0.038	0.069	0.032	0.145	0.284	0.051	0.620
UAE A60 1:30	0.065	0.103	0.055	0.191	0.458	0.089	0.958
UAE A80 1:30	0.049	0.079	0.040	0.166	0.306	0.064	0.703
UAE A100 1:30	0.026	0.049	0.023	0.089	0.188	0.036	0.412
MAE A40 1:15	0.151	0.272	0.125	0.570	1.118	0.199	2.435
MAE A60 1:15	0.250	0.453	0.206	0.850	1.846	0.328	3.929
MAE A80 1:15	0.180	0.330	0.150	0.687	1.342	0.238	3.417
MAE A100 1:15	0.099	0.180	0.081	0.375	0.732	0.129	1.594
MAE A40 1:30	0.116	0.211	0.095	0.442	0.860	0.153	1.875
MAE A60 1:30	0.306	0.400	0.214	0.715	1.728	0.339	3.647
MAE A80 1:30	0.179	0.294	0.152	0.619	1.138	0.238	2.618
MAE A100 1:30	0.079	0.154	0.071	0.275	0.581	0.111	1.272

## Data Availability

Not applicable.

## Supplementary Materials

The following supporting information can be downloaded at: www.mdpi.com/article/10.3390/ijerph19052709/s1, Figure S1: Concentrations of individual phenolic acids in the extracts obtained with UAE (a–d) and MAE (e–h) with 40%, 60%, 80% and 100% acetone. In the abbreviations used to refer to the series, the values after UAE and MAE indicate the extraction time; Figure S2: Correlations between TPC, TFC, phenolic acid content, antioxidant activities and extraction parameters with UAE (a,b) and MAE (c,d) at the solid-to-solvent ratio of 1:15 and 1:30 (*w*:*v*), respectively. Positive correlations are displayed in blue and negative correlations in red. Color intensity and the size of the circles are proportional to the correlation coefficients. Values of Spearman’s rank correlation coefficient above 0.6 are described as a strong correlation; t–extraction time, C–acetone concentration; Table S1: Antioxidant activities of the extracts obtained with UAE and MAE with 40%, 80% and 100% acetone. In the abbreviations used to refer to the series, the values after UAE and MAE indicate the extraction time.

## References

[1] Fito J., Tefera N., Kloos H., Van Hulle S.W. (2019). Physicochemical properties of the sugar industry and ethanol distillery wastewater and their impact on the environment. Sugar Tech.

[2] Klopfenstein T.J., Erickson G.E., Bremer V.R. (2008). Board-Invited Review: Use of distillers by-products in the beef cattle feeding industry. J. Anim. Sci..

[3] Caruso M.C., Braghieri A., Capece A., Napolitano F., Romano P., Galgano F., Altieri G., Genovese F. (2019). Recent updates on the use of agro-food waste for biogas production. Appl. Sci..

[4] Vinatoru M., Mason T.J., Calinescu I. (2017). Ultrasonically assisted extraction (UAE) and microwave assisted extraction (MAE) of functional compounds from plant materials. TrAC-Trend Anal. Chem..

[5] Kumar N., Goel N. (2019). Phenolic acids: Natural versatile molecules with promising therapeutic applications. Biotechnol. Rep..

[6] Farcas A., Dretcanu G., Pop T.D., Enaru B., Socaci S., Diaconeasa Z. (2021). Cereal processing by-products as rich sources of phenolic compounds and their potential bioactivities. Nutrients.

[7] de Araujo F.F., de Paulo Farias D., Neri-Numa I.A., Pastore G.M. (2020). Polyphenols and their applications: An approach in food chemistry and innovation potential. Food Chem..

[8] Smuda S.S., Mohsen S.M., Olsen K., Aly M.H. (2018). Bioactive compounds and antioxidant activities of some cereal milling by-products. J. Food Sci. Technol..

[9] Awika J.M., Rooney L.W., Wu X., Prior R.L., Cisneros-Zevallos J. (2003). Screening methods to measure antioxidant activity of sorghum (sorghum bicolor) and sorghum products. J. Agric. Food Chem..

[10] Granados-Guzman G., Salazar-Aranda R., Garza-Tapia M., Castro-Rios R., Waksman de Torres N. (2017). Optimization and validation of two high-throughput methods indicating antiradical activity. Curr. Anal. Chem..

[11] Yu L., Nanguet A.L., Beta T. (2013). Comparison of antioxidant properties of refined and whole wheat flour and bread. Antioxidants.

[12] Vilkhu K., Mawson R., Simons L., Bates D. (2008). Applications and opportunities for ultrasound assisted extraction in the food industry—A review. Innov. Food Sci. Emerg. Technol..

[13] Khan T., Zahid M., Asim M., Hussan S., Iqbal Z., Choudhary M.I., Ahmad V.U. (2002). Pharmacological activities of crude acetone extract and purified constituents of Salvia moorcraftiana Wall. Phytomedicine.

[14] Sasmal S., Majumdar S., Gupta M., Mukherjee A., Mukherjee P.K. (2012). Pharmacognostical, phytochemical and pharmacological evaluation for the antipyretic effect of the seeds of Saraca asoca Roxb. Asian Pac. J. Trop. Biomed..

[15] Moreira M.M., Morais S., Barros A.A., Delerue-Matos C., Guido L.F. (2012). A novel application of microwave-assisted extraction of polyphenols from brewer’s spent grain with HPLC-DAD-MS analysis. Anal. Bioanal. Chem..

[16] Guido L.F., Moreira M.M. (2017). Techniques for extraction of brewer’s spent grain polyphenols: A review. Food Bioprocess. Technol..

[17] Nayak B., Dahmoune F., Moussi K., Remini H., Dairi S., Aoun O., Khodir M. (2015). Comparison of microwave, ultrasound and accelerated-assisted solvent extraction for recovery of polyphenols from Citrus sinensis peels. Food Chem..

[18] Cherif M.M., Grigorakis S., Halahlah A., Loupassaki S., Makris D.P. (2020). High-efficiency extraction of phenolics from wheat waste biomass (bran) by combining deep eutectic solvent, ultrasound-assisted pretreatment and thermal treatment. Environ. Process..

[19] Vu H.T., Scarlett C.J., Vuong Q.V. (2019). Maximising recovery of phenolic compounds and antioxidant properties from banana peel using microwave assisted extraction and water. J. Food Sci. Technol..

[20] Wang L., Wang A. (2008). Adsorption properties of congo red from aqueous solution onto N,O-carboxymethyl-chitosan. Bioresour. Technol..

[21] Kim K.H., Tsao R., Yang R., Cui S.W. (2006). Phenolic acid profiles and antioxidant activities of wheat bran extracts and the effect of hydrolysis conditions. Food Chem..

[22] Singletion V.L., Orthofer R., Lamuela-Raventos R.M. (1999). 14 Analysis of total phenols and other oxidation substrates and antioxidants by means of Folin-Ciocalteu reagent. Methods Enzymol..

[23] Quettier-Deleu C., Gressier B., Vesseur J., Dine E., Brunet C., Luyckx M., Cazin M., Cazin J.C., Bailleul F. (2000). Phenolic compounds and antioxidant activities of buckwheat (*Fagopyrum esculentum* Moench) hulls and flour. J. Ethnopharm..

[24] Chiremba C., Taylor J.R.N., Rooney L.W., Beta T. (2012). Phenolic acid content of sorghum and maize cultivars varying in hardness. Food Chem..

[25] Re R., Pellegrini N., Proteggente A., Pannala A., Yang M., Rice-Evans C. (1999). Antioxidant activity applying an improved ABTS radical cation decolourisation assay. Free Radic. Biol. Med..

[26] Moure A., Cruz J.M., Franco D., Domıinguez J.M., Sineiro J., Domínguez H., Nunez M.J., Parajó J.C. (2001). Natural antioxidants from residual sources. Food Chem..

[27] Benzie I.F., Strain J.J. (1996). The ferric reducing ability of plasma (FRAP) as a measure of “antioxidant power”: The FRAP assay. Anal. Biochem..

[28] Xie P., Huang L., Zhang C., Deng Y., Wang X., Cheng J. (2019). Enhanced extraction of hydroxytyrosol, maslinic acid and oleanolic acid from olive pomace: Process parameters, kinetics and thermodynamics, and greenness assessment. Food Chem..

[29] Singh B., Singh N., Thakur S., Kaur A. (2017). Ultrasound assisted extraction of polyphenols and their distribution in whole mung bean, hull and cotyledon. J. Food Sci. Technol..

[30] Kwaw E., Ma Y., Tchabo W., Apaliya M.T., Sackey A.S., Wu M., Xiao L. (2018). Impact of ultrasonication and pulsed light treatments on phenolics concentration and antioxidant activities of lactic-acid-fermented mulberry juice. LWT-Food Sci. Technol..

[31] Marszałek K., Mitek M., Skąpska S. (2015). Effect of continuous flow microwave and conventional heating on the bioactive compounds, colour, enzymes activity, microbial and sensory quality of strawberry purée. Food Bioprocess Technol..

[32] Qiao L., Sun Y., Chen R., Fu Y., Zhang W., Li X., Chen J., Shen Y., Ye X. (2014). Sonochemical effects on 14 flavonoids common in citrus: Relation to stability. PLoS ONE.

[33] Vu H.T., Scarlett C.J., Vuong Q.V. (2016). Effects of drying conditions on physicochemical and antioxidant properties of banana (Musa cavendish) Peels. Dry. Technol..

[34] Di Mattia D.C., Sacchetti G., Mastrocola D., Sarker D.K., Pittia P. (2010). Surface properties of phenolic compounds and their influence on the dispersion degree and oxidative stability of olive oil O/W emulsions. Food Hydrocoll..

[35] Zuorro A., Iannone A., Lavecchia R. (2019). Water–organic solvent extraction of phenolic antioxidants from brewers’ spent grain. Processes.

[36] Mohammadi M., Alaei M., Bajalan I. (2016). Phytochemical screening, total phenolic and flavonoid contents and antioxidant activity of Anabasis setifera and Salsola tomentosa extracted with different extraction methods and solvents. Orient. Pharm. Exp. Med..

[37] Hansen C.M. (2007). Hansen Solubility Parameters. A User’s Handbook.

[38] Zollmann M., Robin A., Prabhu M., Polikovsky M., Gillis A., Greiserman S., Golberg A. (2019). Green technology in green macroalgal biorefineries. Phycologia.

[39] Friedman M., Jürgens H.S. (2000). Effect of pH on the stability of plant phenolic compounds. J. Agric. Food Chem..

[40] Chen Y., Ma Y., Dong L., Jia X., Liu L., Huang F., Chi J., Xiao J., Zhang M., Zhang R. (2019). Extrusion and fungal fermentation change the profile and antioxidant activity of free and bound phenolics in rice bran together with the phenolic bioaccessibility. LWT-Food Sci. Technol..

[41] Schwab W., Davidovich-Rikanati R., Lewinsohn E. (2008). Biosynthesis of plant-derived flavor compounds. Plant J..

[42] Skrajda-Brdak M., Konopka I., Tańska M., Czaplicki S. (2019). Changes in the content of free phenolic acids and antioxidative capacity of wholemeal bread in relation to cereal species and fermentation type. Eur. Food Res. Technol..

[43] Maruf A.A., Lip H., Wong H., O’Brien P.J. (2015). Protective effects of ferulic acid and related polyphenols against glyoxal- or methylglyoxal-induced cytotoxicity and oxidative stress in isolated rat hepatocytes. Chem. Biol. Interact..

[44] Sibhatu H.K., Jabasingh S.A., Yimam A., Ahmed S. (2021). Ferulic acid production from brewery spent grains, an agro-industrial waste. LWT-Food Sci. Technol..

[45] Timokhin V.I., Regner M., Motagamwala A.H., Sener C., Karlen S.D., Dumesic J.A., Ralph J. (2020). Production of p-coumaric acid from corn GVL-lignin. ACS Sustain. Chem. Eng..

[46] Ravichandran K., Ahmed A.R., Knorr D., Smetanska I. (2012). The effect of different processing methods on phenolic acid content and antioxidant activity of red beet. Int. Food Res. J..

[47] Chen C. (2016). Sinapic acid and its derivatives as medicine in oxidative stress-induced diseases and aging. Oxid. Med. Cell. Longev..

[48] Mehmood A., Ishaq M., Zhao L., Yaqoob S., Safdar B., Nadeem M., Munir M., Wang C. (2019). Impact of ultrasound and conventional extraction techniques on bioactive compounds and biological activities of blue butterfly pea flower (*Clitoria ternatea* L.). Ultrason. Sonochem..

[49] Kikuzaki H., Hisamoto M., Hirose K., Akiyama K., Taniguchi H. (2002). Antioxidant properties of ferulic acid and its related compounds. J. Agric. Food Chem..

[50] Strazisar M., Andrensek S., Smidovnik A. (2008). Effect of β-cyclodextrin on antioxidant activity of coumaric acids. Food Chem..

[51] Hernandez J.E., Edyvean R.G.J. (2008). Inhibition of biogas production and biodegradability by substituted phenolic compounds in anaerobic sludge. J. Hazard. Mater..

[52] Kerkel F., Brock D., Touraud D., Kunz W. (2021). Stabilisation of biofuels with hydrophilic, natural antioxidants solubilised by glycerol derivatives. Fuel.

[53] Soto M.L., Moure A., Dominguez H., Parajó J.C. (2011). Recovery, concentration and purification of phenolic compounds by adsorption: A review. J. Food Eng..

