# Supplementary material for: Processing of Distillery Stillage to Recover Phenolic Compounds with Ultrasound-Assisted and Microwave-Assisted Extractions
Source: Int J Environ Res Public Health. 2022 Feb 25;19(5):2709. doi: 10.3390/ijerph19052709 (PMC8910419; doi:10.3390/ijerph19052709)
Supplement: Supplementary file 1 [file ijerph-19-02709-s001.zip › ijerph-1593984-supplementary.pdf]

Processing of distillery stillage to recover phenolic compounds with ultrasound-assisted and microwave-assisted extractions

Wioleta Mikucka[a*], Magdalena Zielinska[a], Katarzyna Bulkowska[a], Izabela Witonska[b]
[a]University of Warmia and Mazury in Olsztyn, Faculty of Geoengineering, Department of Environmental Biotechnology, Sloneczna St. 45G, 10-709 Olsztyn, Poland; wioleta.mikucka@uwm.edu.pl (W.M.); magdalena.zielinska@uwm.edu.pl (M.Z.); katarzyna.bulkowska@uwm.edu.pl (K.B.)
[b]Lodz University of Technology, Faculty of Chemistry, Institute of General and Ecological Chemistry, Zeromskiego St. 116, 90-924 Lodz, Poland; izabela.witonska@p.lodz.pl
*Corresponding author: wioleta.mikucka@uwm.edu.pl

Supplementary Materials

(a)

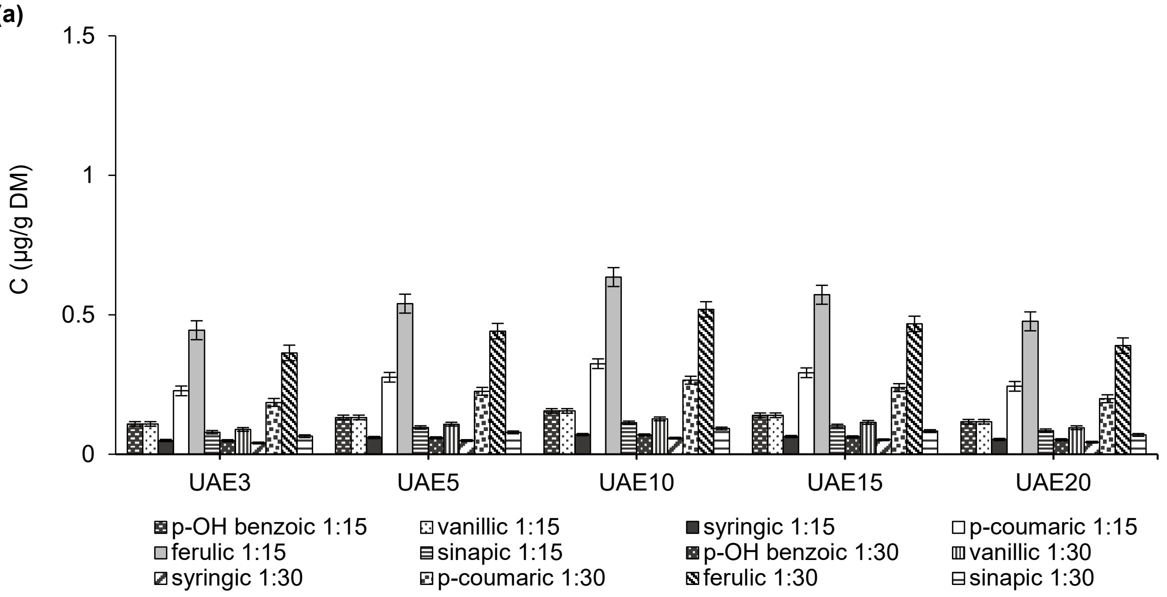

(b)

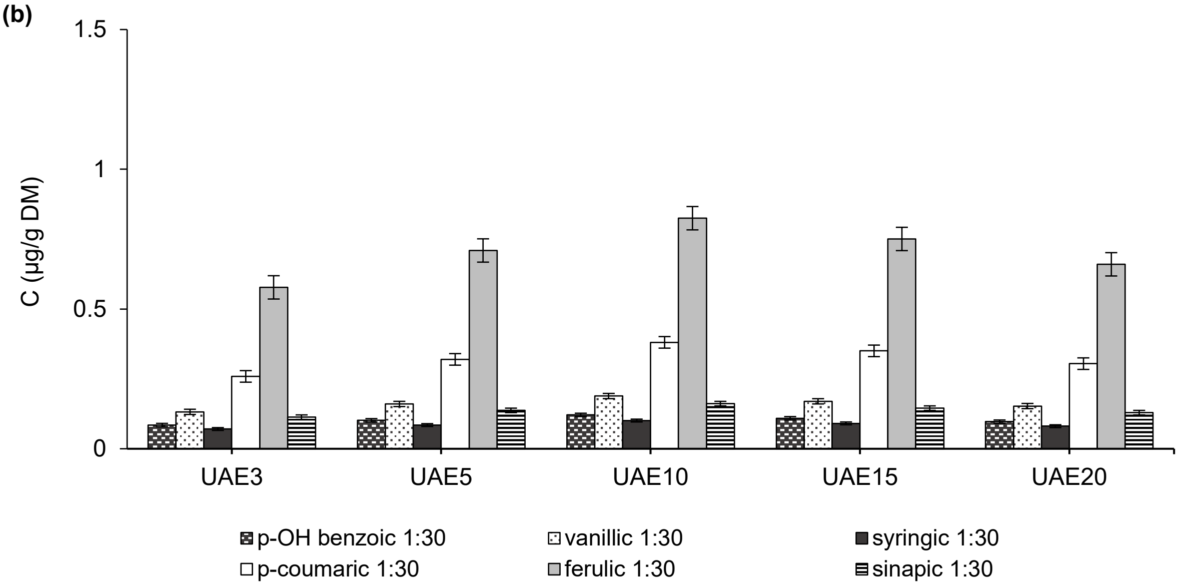

(c)

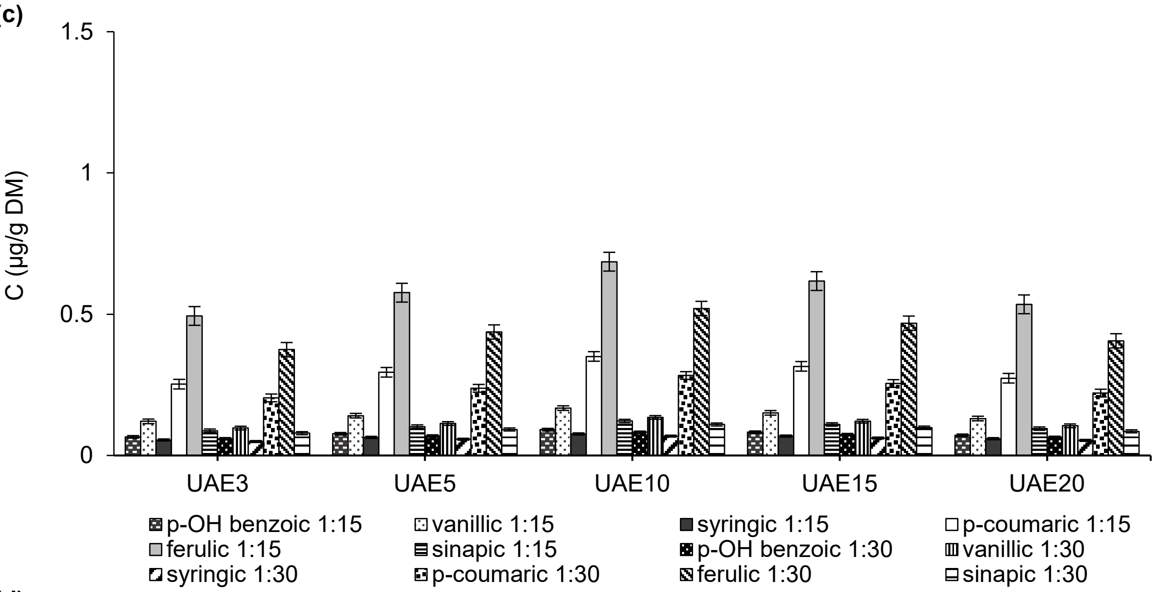
C (µg/g DM)
1.5
1
0.5
0
UAE3
UAE5
UAE10
UAE15
UAE20
p-OH benzoic 1:15
vanillic 1:15
syringic 1:15
p-coumaric 1:15
ferulic 1:15
sinapic 1:15
p-OH benzoic 1:30
vanillic 1:30
syringic 1:30
p-coumaric 1:30
ferulic 1:30
sinapic 1:30

(d)

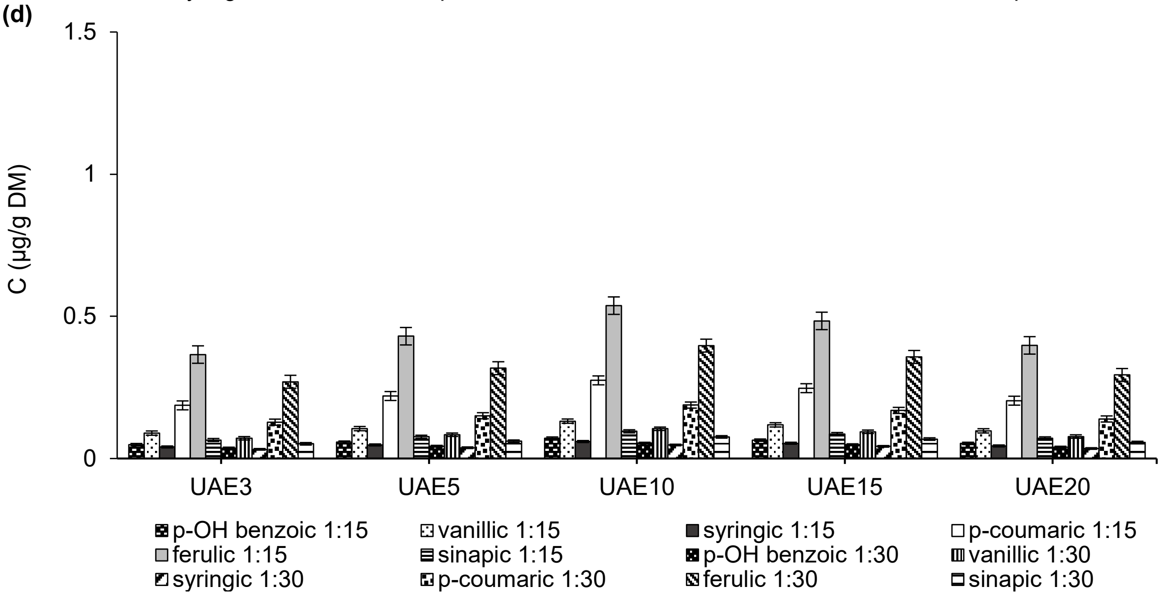
C (µg/g DM)
1.5
1
0.5
0
UAE3
UAE5
UAE10
UAE15
UAE20
p-OH benzoic 1:15
vanillic 1:15
syringic 1:15
p-coumaric 1:15
ferulic 1:15
sinapic 1:15
p-OH benzoic 1:30
vanillic 1:30
syringic 1:30
p-coumaric 1:30
ferulic 1:30
sinapic 1:30

(e)

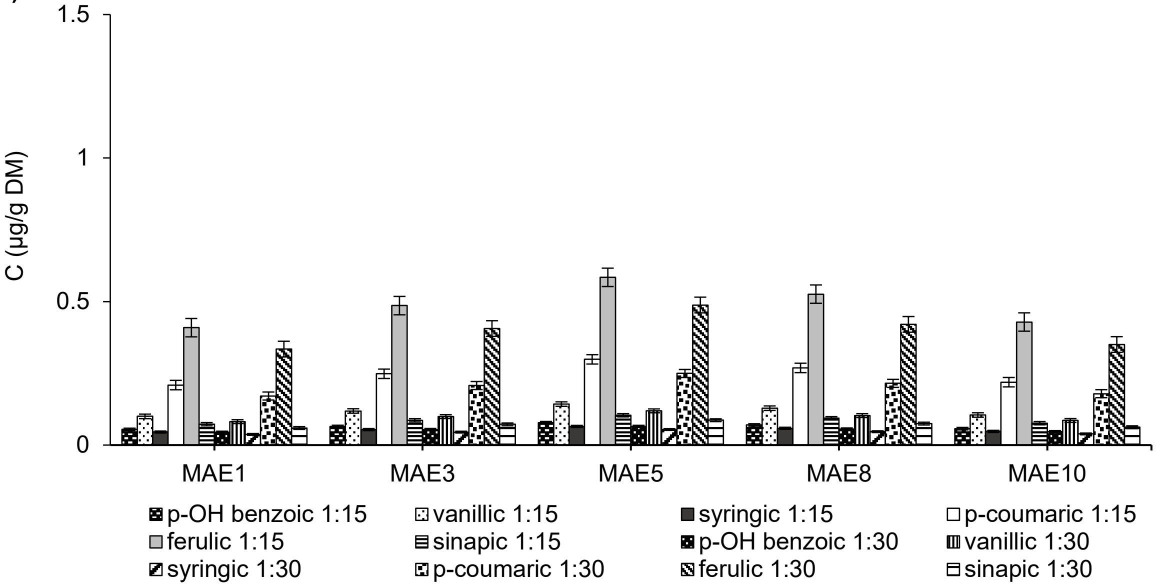
C (µg/g DM)
1.5
1
0.5
0
MAE1
MAE3
MAE5
MAE8
MAE10
p-OH benzoic 1:15
vanillic 1:15
syringic 1:15
p-coumaric 1:15
ferulic 1:15
sinapic 1:15
p-OH benzoic 1:30
vanillic 1:30
syringic 1:30
p-coumaric 1:30
ferulic 1:30
sinapic 1:30

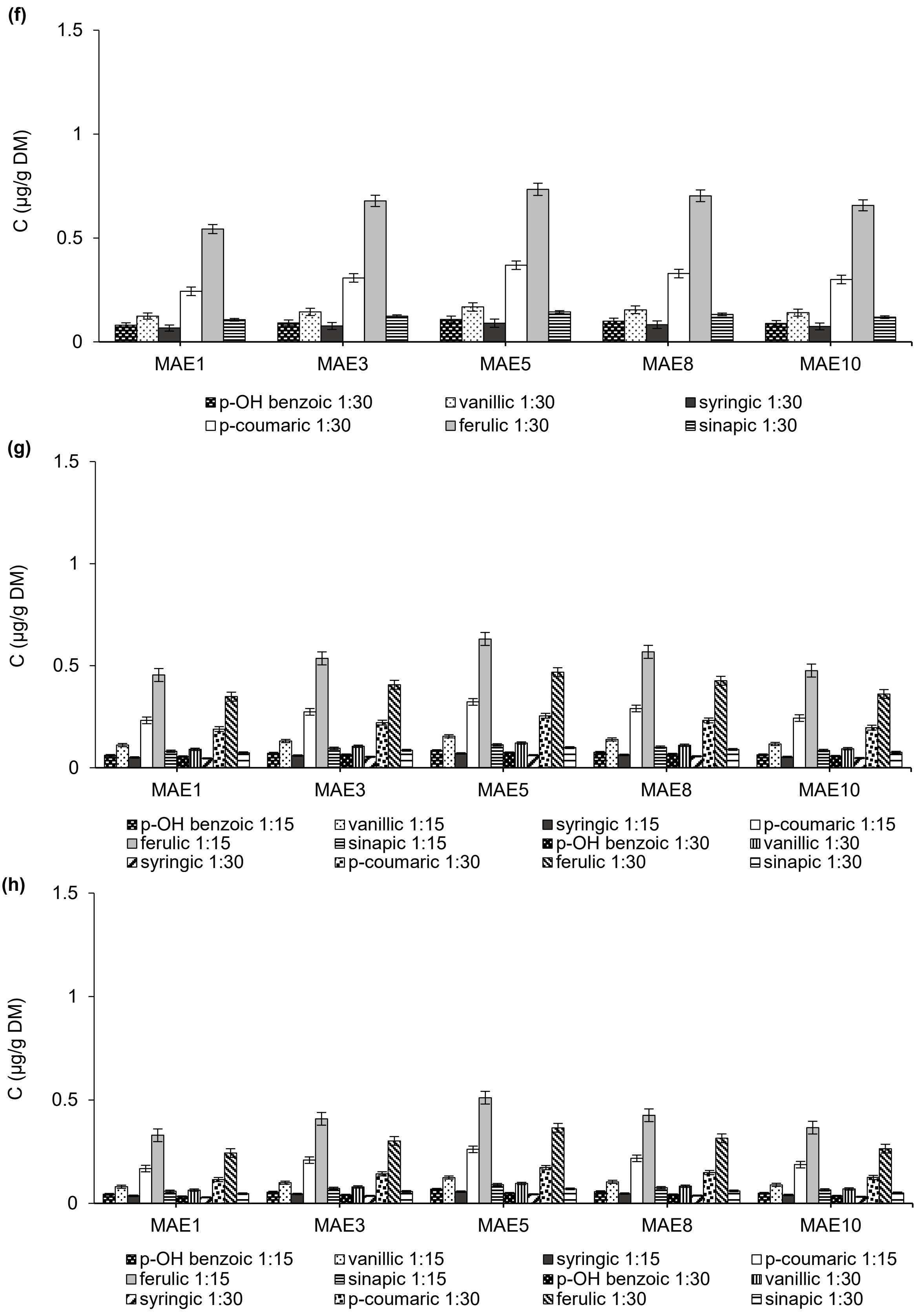

Figure S1. Concentrations of individual phenolic acids in the extracts obtained with UAE **(a, b, c, d**) and MAE **(e, f, g, h)** with 40%, 60%, 80% and 100% acetone. In the abbreviations used to refer to the series, the values after UAE and MAE indicate the extraction time.

(a)

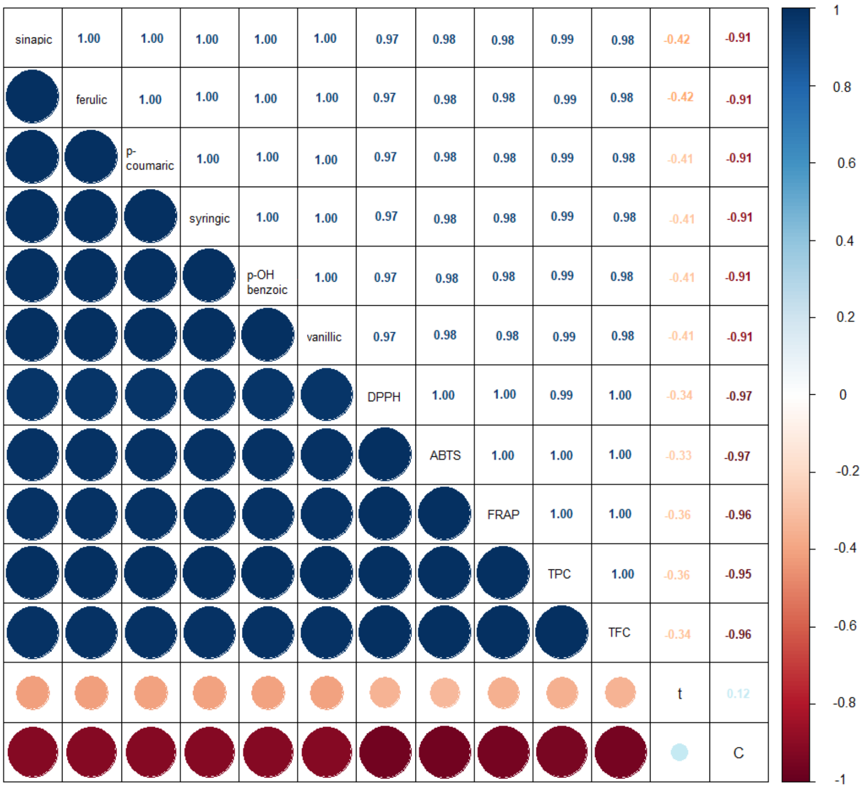
sinapic 1.00 1.00 1.00 1.00 1.00 0.97 0.98 0.98 0.99 0.98 -0.42 -0.91
ferulic 1.00 1.00 1.00 1.00 0.97 0.98 0.98 0.99 0.98 -0.42 -0.91
p-coumaric 1.00 1.00 1.00 0.97 0.98 0.98 0.99 0.98 -0.41 -0.91
syringic 1.00 1.00 0.97 0.98 0.98 0.99 0.98 -0.41 -0.91
p-OH benzoic 1.00 0.97 0.98 0.98 0.99 0.98 -0.41 -0.91
vanillic 0.97 0.98 0.98 0.99 0.98 -0.41 -0.91
DPPH 1.00 1.00 0.99 1.00 -0.34 -0.97
ABTS 1.00 1.00 1.00 -0.33 -0.97
FRAP 1.00 1.00 -0.36 -0.96
TPC 1.00 -0.36 -0.95
TFC -0.34 -0.96
t 0.12
C
1
0.8
0.6
0.4
0.2
0
-0.2
-0.4
-0.6
-0.8
-1

(b)

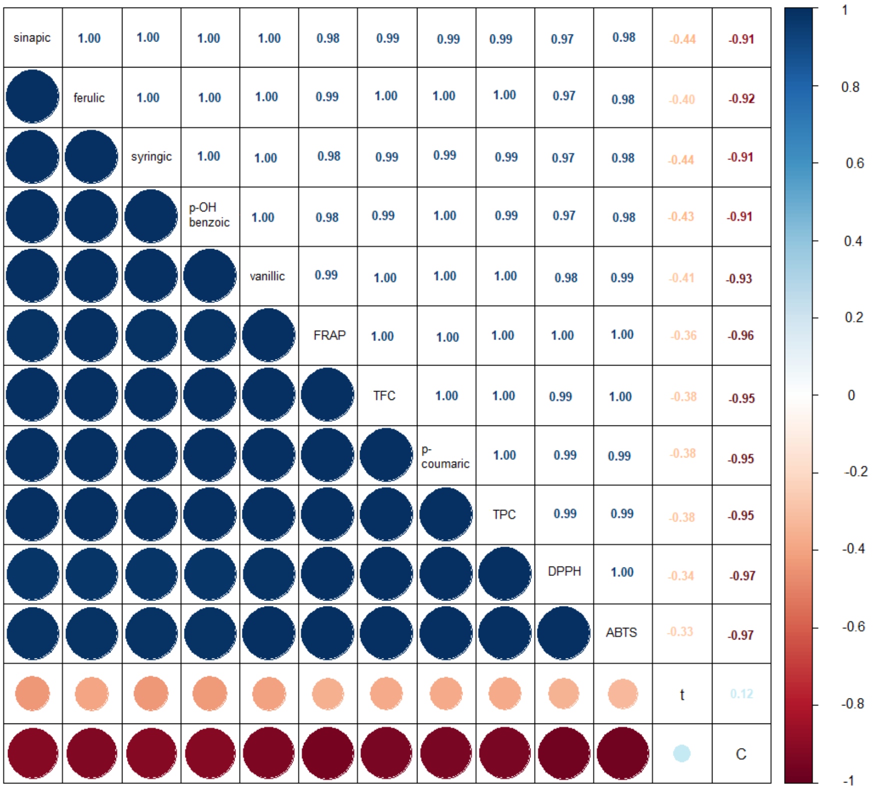
sinapic 1.00 1.00 1.00 1.00 0.98 0.99 0.99 0.99 0.97 0.98 -0.44 -0.91
ferulic 1.00 1.00 1.00 0.99 1.00 1.00 1.00 0.97 0.98 -0.40 -0.92
syringic 1.00 1.00 0.98 0.99 0.99 0.99 0.97 0.98 -0.44 -0.91
p-OH benzoic 1.00 0.98 0.99 1.00 0.99 0.97 0.98 -0.43 -0.91
vanillic 0.99 1.00 1.00 1.00 0.98 0.99 -0.41 -0.93
FRAP 1.00 1.00 1.00 1.00 1.00 -0.36 -0.96
TFC 1.00 1.00 0.99 1.00 -0.38 -0.95
p-coumaric 1.00 0.99 0.99 -0.38 -0.95
TPC 0.99 0.99 -0.38 -0.95
DPPH 1.00 -0.34 -0.97
ABTS -0.33 -0.97
t 0.12
C
1
0.8
0.6
0.4
0.2
0
-0.2
-0.4
-0.6
-0.8
-1

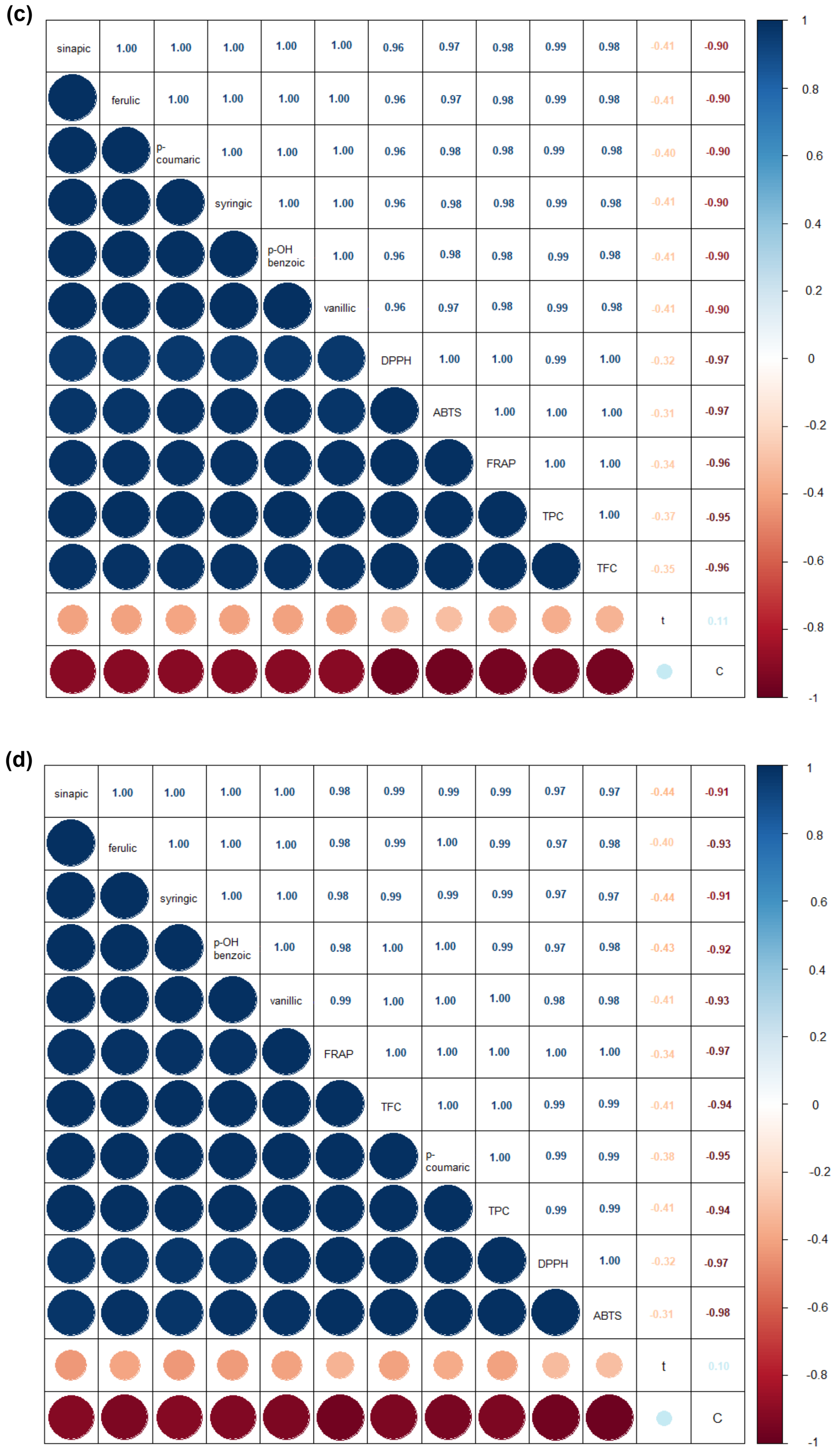

Figure S2. Correlations between TPC, TFC, phenolic acid content, antioxidant activities and extraction parameters with UAE **(a, b)** and MAE **(c, d)** at the solid-to-solvent ratio of 1:15 and 1:30 (w:v), respectively. Positive correlations are displayed in blue and negative correlations in red. Color intensity and the size of the circles are proportional to the correlation coefficients. Values of Spearman's rank correlation coefficient above 0.6 are described as a strong correlation; t – extraction time, C – acetone concentration

Table S1. Antioxidant activities of the extracts obtained with UAE and MAE with 40%, 80% and 100% acetone. In the abbreviations used to refer to the series, the values after UAE and MAE indicate the extraction time.

Type of extraction	Antioxidant activity			Type of extraction	Antioxidant activity		
	DPPH (μmol TE/g DM)	FRAP (μmol $FeSO_4$/g DM)	ABTS (μmol TE/g DM)		DPPH (μmol TE/g DM)	FRAP (μmol $FeSO_4$/g DM)	ABTS (μmol TE/g DM)
UAE3 A40 1:15	3.61±0.57	2.11±0.23	6.44±0.68	MAE1 A40 1:15	3.00±0.62	1.75±0.27	5.35±0.87
UAE3 A80 1:15	4.09±0.48	2.30±0.28	6.42±0.66	MAE1 A80 1:15	3.39±0.55	1.91±0.23	5.33±0.86
UAE3 A100 1:15	2.48±0.37	1.37±0.15	4.55±0.58	MAE1 A100 1:15	2.06±0.42	1.14±0.19	3.78±0.76
UAE5 A40 1:15	4.39±0.41	2.56±0.27	7.82±0.78	MAE3 A40 1:15	3.64±0.48	2.12±0.24	6.49±0.55
UAE5 A80 1:15	4.77±0.44	2.69±0.26	7.49±0.83	MAE3 A80 1:15	3.96±0.51	2.23±0.22	6.22±0.49
UAE5 A100 1:15	2.92±0.29	1.62±0.34	5.36±0.73	MAE3 A100 1:15	2.48±0.39	1.37±0.11	4.55±0.63
UAE10 A40 1:15	5.16±0.62	3.01±0.42	9.20±0.94	MAE5 A40 1:15	4.39±0.35	2.56±0.26	7.82±0.72
UAE10 A80 1:15	5.68±0.66	3.20±0.40	8.92±0.91	MAE5 A80 1:15	4.71±0.43	2.66±0.31	7.40±0.77
UAE10 A100 1:15	3.65±0.34	2.02±0.29	6.70±0.61	MAE5 A100 1:15	3.03±0.31	1.68±0.29	5.56±0.63
UAE15 A40 1:15	4.64±0.43	2.71±0.25	8.28±0.76	MAE8 A40 1:15	3.72±0.28	2.17±0.24	6.62±0.45
UAE15 A80 1:15	5.11±0.61	2.88±0.23	8.03±0.83	MAE8 A80 1:15	4.09±0.32	2.30±0.11	6.42±0.59
UAE15 A100 1:15	3.28±0.35	1.82±0.16	6.03±0.56	MAE8 A100 1:15	2.63±0.46	1.45±0.14	4.82±0.29
UAE20 A40 1:15	3.87±0.38	2.26±0.19	6.90±0.71	MAE10 A40 1:15	3.21±0.25	1.87±0.26	5.73±0.47
UAE20 A80 1:15	4.43±0.42	2.50±0.21	6.96±0.72	MAE10 A80 1:15	3.72±0.32	2.10±0.24	5.84±0.44
UAE20 A100 1:15	2.70±0.19	1.49±0.13	4.96±0.55	MAE10 A100 1:15	2.27±0.29	1.26±0.22	4.16±0.36
UAE3 A40 1:30	3.32±0.27	1.94±0.18	5.93±0.59	MAE1 A40 1:30	2.76±0.33	1.61±0.08	4.92±0.64
UAE3 A80 1:30	3.76±0.35	2.12±0.16	5.91±0.61	MAE1 A80 1:30	3.05±0.28	1.72±0.14	4.78±0.59
UAE3 A100 1:30	2.28±0.29	1.26±0.09	4.19±0.44	MAE1 A100 1:30	1.85±0.19	1.02±0.09	3.39±0.38
UAE5 A40 1:30	4.04±0.43	2.35±0.22	7.19±0.38	MAE3 A40 1:30	3.35±0.23	1.95±0.13	5.97±0.42
UAE5 A80 1:30	4.39±0.46	2.47±0.13	6.89±0.63	MAE3 A80 1:30	3.77±0.26	2.13±0.18	5.93±0.78
UAE5 A100 1:30	2.68±0.24	1.49±0.11	4.93±0.52	MAE3 A100 1:30	2.31±0.18	1.28±0.24	4.24±0.55
UAE10 A40 1:30	4.75±0.60	2.77±0.16	8.46±0.68	MAE5 A40 1:30	4.08±0.44	2.38±0.28	7.28±0.49
UAE10 A80 1:30	5.22±0.43	2.94±0.23	8.20±0.73	MAE5 A80 1:30	4.33±0.46	2.44±0.25	6.81±0.38

UAE10 A100 1:30	3.36±0.34	1.86±0.17	6.16±0.69	MAE5 A100 1:30	2.78±0.55	1.54±0.15	5.11±0.69
UAE15 A40 1:30	4.27±0.32	2.49±0.28	7.62±0.75	MAE8 A40 1:30	3.46±0.37	2.02±0.17	6.17±0.54
UAE15 A80 1:30	4.70±0.46	2.65±0.34	7.38±0.78	MAE8 A80 1:30	3.81±0.31	2.15±0.09	5.98±0.23
UAE15 A100 1:30	3.02±0.26	1.67±0.21	5.54±0.52	MAE8 A100 1:30	2.51±0.25	1.39±0.16	4.60±0.39
UAE20 A40 1:30	3.56±0.22	2.08±0.29	6.35±0.33	MAE10 A40 1:30	2.92±0.21	1.70±0.18	5.21±0.44
UAE20 A80 1:30	4.07±0.38	2.30±0.31	6.40±0.29	MAE10 A80 1:30	3.42±0.16	1.93±0.21	5.38±0.62
UAE20 A100 1:30	2.48±0.36	1.38±0.05	4.56±0.34	MAE10 A100 1:30	2.06±0.13	1.17±0.08	3.87±0.41
